# CSFINet-Based Multimodal MRI Image Segmentation and Survival Prediction

**DOI:** 10.3390/diagnostics16193210

**Published:** 2026-10-02

**Authors:** Jia-Lien Hsu, Pei-Yu Yu

**Affiliations:** Department of Computer Science and Information Engineering, Fu Jen Catholic University, New Taipei City 24205, Taiwan; dannyyo91@gmail.com

**Keywords:** brain tumor MRI, multimodal analysis, CSFINet, overall survival prediction, ablation study, explainable artificial intelligence (xAI)

## Abstract

**Background**: Brain tumors are highly lethal malignant neoplasms, for which accurate tumor segmentation and survival-day prediction are critical to treatment planning and prognostic assessment. This study establishes an exploratory two-stage analytical framework spanning multimodal MRI tumor segmentation and survival-day prediction. **Methods**: The study is based on the BraTS 2020 multimodal MRI dataset, employing four imaging modalities—T1, T1ce, T2, and FLAIR—with patient age and extent-of-resection status as candidate clinical features. The 235 cases with survival labels were split at the patient level (188 training, 47 test; single 80:20 split). In the first stage, CSFINet is used for brain tumor segmentation; in the second stage, the resulting binary whole-tumor mask, age, and resection status serve as inputs for survival-day prediction. The survival models are trained on the ground-truth whole-tumor masks but evaluated on the masks predicted by CSFINet, and the survival training runs were not seeded. Four models—image-only, image with age, image with resection status, and image with both—are constructed for ablation experiments, and Gradient SHAP is introduced for interpretability analysis. **Results**: CSFINet achieves a Mean Dice, Mean Precision, and Mean Recall of 0.7849±0.1937, 0.8739±0.1978, and 0.7480±0.1936, compared with 0.5084, 0.8676, and 0.3822 for a U-Net baseline trained in this study under an identical configuration. The gains are in Dice and recall; the precision difference (+0.0063, 95% CI −0.0601 to 0.1086) is not distinguishable from zero. This comparison is internal to the present study; published BraTS 2020 results were obtained under different evaluation protocols and are not directly comparable. The model combining imaging features with age yields the lowest error, achieving a mean absolute error of 251.69 days (95% CI [186.56, 336.79])—lower than the 279.41 days obtained by the image-only model—whereas incorporating resection status does not lead to further improvement. **Conclusions**: CSFINet improves whole-tumor segmentation relative to this identically configured U-Net baseline—not relative to the published state of the art—and the inclusion of age was associated with a lower survival-day prediction error in this study. The survival findings are exploratory and cohort-specific: an MAE of 252 days is roughly 57% of the reported median survival of glioblastoma and is not clinically actionable, and the web interface is a research prototype. This study establishes an integrated analytical pipeline spanning tumor segmentation, survival-day prediction, and result visualization, serving as a preliminary reference for brain tumor image analysis and prognostic assessment. These are exploratory, single-cohort associations obtained from BraTS 2020; they do not establish a causal relationship between the clinical variables and survival, and they have not been externally validated.

## 1. Introduction

This section first outlines the clinical significance of glioma in diagnosis and prognostic assessment, along with the utility of multimodal MRI and clinical data for brain tumor analysis. The challenges inherent in tumor segmentation, overall survival prediction, and model interpretability are then examined. Finally, a two-stage multimodal brain tumor analysis framework based on the Cross-scale Feature Interaction Network (CSFINet) [1] is presented, in which Gradient SHAP is employed to characterize the basis of the model’s segmentation decisions.

### 1.1. Research Background and Motivation

Glioma is one of the most common primary tumors of the central nervous system and can be categorized into different types and grades of malignancy according to its histopathological characteristics. Among these, glioblastoma (GBM) is highly infiltrative and aggressive, with a five-year relative survival rate below 10% [2]. In current clinical practice, accurate tumor assessment extends beyond anatomical lesion delineation to survival estimation, which is of considerable importance for the formulation of individualized treatment strategies [3,4].

Clinical diagnosis currently relies primarily on magnetic resonance imaging (MRI), including the four modalities of T1, T1ce, T2, and FLAIR. Different modalities reveal distinct imaging characteristics of tumor tissue, edematous regions, and enhancing regions, and are therefore widely used for brain tumor segmentation and analysis. However, when predicting a patient’s overall survival (OS), imaging features alone are often insufficient to fully characterize prognosis. Clinical studies have indicated that patient age and the extent of resection may both influence survival outcomes in patients with glioblastoma, and both are regarded as important prognostic factors [5]. Accordingly, building upon tumor image analysis, this study adopts age and resection status as candidate clinical features and establishes a two-stage analysis pipeline that proceeds from tumor segmentation to overall survival prediction, with ablation experiments conducted to evaluate the auxiliary value of the different clinical features.

The extent of surgical resection is among the most consistently reported prognostic factors in glioblastoma, and the quantity that carries the prognostic information is the residual tumor volume rather than the surgeon-reported category. Lacroix et al. [6] reported, in 416 patients with glioblastoma multiforme, that resection of at least 98% of the enhancing tumor volume was associated with significantly longer median survival (13.0 vs. 8.8 months). Sanai et al. [7] subsequently identified a stepwise survival benefit beginning at an extent of resection of approximately 78%. More recent work has shown that maximal resection of both contrast-enhanced and non-contrast-enhanced tumor is associated with longer survival, with the strength of the association differing across molecular subgroups [8]. The RANO resect group has proposed and prognostically validated a classification of residual disease based on the volumes of residual contrast-enhanced and non-contrast-enhanced tumor [9]. BraTS 2020, however, provides only the categorical label—gross total resection (GTR), subtotal resection (STR), or unknown/unlabeled (NA)—and distributes neither postoperative imaging nor residual tumor volumes. The three-level categorical encoding used in the present study is therefore imposed by the available data and is expected to attenuate any association between the extent of resection and survival; the resulting interpretive limitations are discussed in Section 4.2.

### 1.2. Research Problems and Challenges

The proposed brain MRI analysis system addresses three principal challenges: tumor image segmentation, clinical data integration, and model interpretability. Although U-Net has been widely applied to medical image processing, its feature propagation mechanism may inadequately integrate shallow-level spatial detail with deep-level semantic information when confronted with the scale variation, irregular morphology, and ambiguous boundaries typical of brain tumors, thereby degrading tumor localization and boundary prediction.

In the overall survival prediction task, patient prognosis is influenced not only by tumor imaging characteristics but also, potentially, by clinical factors such as age and resection status. Key challenges include how to transform the binary whole-tumor mask produced in the first stage into three-dimensional image features suitable for model learning, and how to fuse these features with clinical data. The auxiliary value of each clinical feature is then assessed through ablation experiments.

Furthermore, deep learning models are frequently regarded as black boxes in medical imaging applications. When a model provides only a segmentation mask or a prediction result, users cannot readily determine whether its judgment is based on clinically plausible lesion regions. This study therefore employs visualization methods to examine the decision basis of the segmentation model, thereby improving the transparency and trustworthiness of the results.

### 1.3. Research Objectives and Contributions

To address the challenges posed by ambiguous tumor boundaries in brain tumor MRI, the pronounced variation in lesion size, and the influence of multiple clinical factors on survival prognosis, this study aims to establish a two-stage multimodal brain tumor analysis pipeline based on CSFINet. In the first stage, CSFINet is used to segment the tumor regions; through the Patch-wise Interaction Unit (PIU) and the Feature-wise Interaction Unit (FIU), the pipeline strengthens the integration of information across features at different scales, thereby mitigating the loss of spatial detail that conventional convolutional architectures may incur during successive downsampling.

In the second stage, the tumor segmentation results serve as the image input, while patient age and resection status are adopted as candidate clinical features. The model extracts spatial and morphological tumor features via a 3D CNN and encodes individual-level information via a multilayer perceptron (MLP); the resulting representations are fused and used to predict overall survival in days. To assess the auxiliary value of the different clinical features, ablation experiments are conducted using four input configurations: image only, image combined with age, image combined with resection status, and image combined with both age and resection status.

In addition, this study incorporates Gradient SHAP to conduct an interpretability analysis of the image segmentation model, comparing the spatial distribution of the pixel-level attribution results with that of the expert annotations and the model’s predicted regions, in order to examine the image locations on which the model relies when performing tumor segmentation. This analysis serves only as an auxiliary means of examining the model’s decision basis and does not in itself indicate that the model possesses clinical diagnostic capability.

In summary, the main contributions of this study are as follows:

1. We establish a four-modality brain tumor segmentation pipeline centered on CSFINet, which performs pixel-wise classification into background, NCR/NET, ED, and ET, and subsequently merges these classes into a binary whole-tumor mask.
2. We construct overall survival prediction models under four different input configurations and analyze, through ablation experiments, the auxiliary value of age and resection status for prediction performance.
3. We introduce Gradient SHAP for pixel-level attribution analysis in order to examine the decision basis on which the segmentation model relies when identifying tumors.

### 1.4. Related Work

We first outline the evolution of medical image segmentation techniques, from conventional convolutional neural networks to the now-prevalent U-Net architecture and the challenges it faces. We then examine the application of attention mechanisms and the Vision Transformer to the modeling of global information. The section further describes the characteristics of multimodal MRI, the influence of clinical indicators on the survival prediction task, and the definition of the BraTS dataset. Finally, we discuss the necessity of explainable artificial intelligence (xAI) in medical imaging together with the associated theory.

#### 1.4.1. Evolution of Medical Image Segmentation Techniques

The advent of convolutional neural networks (CNNs) marked the transition of medical image analysis from hand-crafted features to a new era of automated feature extraction. In medical image processing, CNNs learn hierarchical spatial features directly from raw data through the alternating operation of convolutional layers, pooling layers, and activation functions. Compared with conventional algorithms, CNNs exhibit translation equivariance and weight sharing [10], conferring strong robustness and representational capacity when processing medical images with regular anatomical structures, such as MRI and CT. Early studies focused primarily on image classification; however, as computational capacity increased, research attention gradually shifted toward the more demanding task of semantic segmentation, which aims to achieve pixel-wise localization and precise delineation of lesion boundaries [11].

Within the development of medical image segmentation, U-Net and its subsequent variants constitute a representative class of encoder–decoder architectures [11,12]. U-Net adopts a symmetric encoder–decoder structure composed of a contracting path and an expansive path. The encoder extracts high-level semantic information through successive convolution and downsampling operations, while the decoder progressively restores the image resolution through upsampling. One of the key design elements of U-Net is the skip connection, which transfers shallow-level spatial features from the encoder to the corresponding decoder layer and fuses them with high-level semantic features. This compensates for the spatial detail that may be lost during downsampling and thereby helps improve lesion localization and boundary segmentation [11].

Although U-Net achieves strong performance across many medical image segmentation tasks, it may nonetheless encounter difficulties when applied to highly heterogeneous lesions such as brain tumors [13]. The subregions in the BraTS data—the tumor core, the peritumoral edema, and the enhancing tumor—differ markedly in size, morphology, and imaging appearance, requiring the model to account simultaneously for local spatial detail and semantic information over a broader spatial extent [14,15].

In recent years, research on the U-Net family of architectures can be broadly divided into two directions. The first strengthens the training pipeline and automates hyperparameter configuration without altering the core architecture. The nnU-Net proposed by Isensee et al. is a representative example: based on the image resolution, voxel spacing, and memory constraints of a given dataset, the method automatically determines the preprocessing scheme, network depth, patch size, and training strategy, and has achieved competitive results in numerous medical image segmentation challenges, including those involving brain tumors. This suggests that the design of the training pipeline may influence segmentation performance no less than the network architecture itself [16]. The second direction modifies the encoder–decoder structure itself, encompassing nested skip connections, attention mechanisms, and Transformer modules. Azad et al. conducted a systematic review of U-Net and its subsequent variants, organizing existing improvements into categories such as enhanced skip connections, bottleneck design, multi-scale fusion, and attention mechanisms, and noted that cross-scale feature integration remains an incompletely resolved problem in medical image segmentation [17].

Moreover, in conventional encoder–decoder architectures, successive downsampling may result in the loss of certain spatial details, and skip connections that directly fuse shallow- and deep-level features do not necessarily capture the interdependencies among features at different scales. Strengthening cross-scale feature interaction and information integration is therefore an important direction for improving the segmentation of complex lesions [1].

#### 1.4.2. Attention Mechanisms and the Vision Transformer

Conventional convolution extracts features within a local receptive field, so associations between distant image regions must be established incrementally across multiple layers. To strengthen a model’s capacity to represent long-range dependencies, non-local operations and attention mechanisms have progressively been adopted in image analysis tasks [18]. Attention mechanisms adjust the weights assigned to different positions or channels according to the importance of the input features. CBAM, for example, combines channel attention and spatial attention, sequentially reweighting the channels and spatial positions of a feature map [19]. In medical image segmentation, Attention U-Net employs attention gates to suppress features unrelated to the target structure and to direct the model’s focus toward relevant image regions [20].

More recently, the Vision Transformer (ViT) has been increasingly applied to medical image segmentation on account of its capacity for global modeling. Whereas convolution operations extract features primarily within a local neighborhood, the Transformer establishes long-range associations among image patches or feature positions through the self-attention mechanism. In Transformer-based medical image segmentation models, the Transformer may serve as the encoder or be combined with a convolutional architecture to extract high-level semantic information. By partitioning an image into multiple patches and processing them as a sequence, such models integrate feature information from different image regions and improve the representation of both local detail and global structure [21].

However, the original ViT performs global self-attention over all image patches, and its computational complexity grows quadratically with image size, which makes it difficult to apply directly to high-resolution medical images. To address this issue, Liu et al. proposed the Swin Transformer, which confines self-attention to local windows through window-based self-attention and establishes cross-window information exchange through a shifted-window mechanism. Combined with its hierarchical feature representation, this design enables the Transformer to be applied to dense prediction tasks in a multi-scale manner analogous to convolutional networks [22]. The CSFINet adopted in this study likewise employs Swin Transformer blocks within its cross-scale feature interaction modules.

In the context of medical image segmentation, Cao et al. proposed Swin-Unet, which replaces all convolutional modules in the U-shaped encoder–decoder with Swin Transformer blocks and substitutes patch-expanding layers for conventional upsampling operations, demonstrating that a pure Transformer architecture can also perform pixel-level segmentation [23]. Zhou et al. proposed nnFormer, which interleaves convolution operations with local and global self-attention within a three-dimensional medical image segmentation architecture, using convolution to capture local detail and self-attention to model longer-range contextual relationships, thereby exploiting the complementary characteristics of both operations [24].

For the brain tumor segmentation task specifically, Wang et al. proposed TransBTS, which employs a three-dimensional convolutional network as the encoder to extract local spatial features, introduces a Transformer at the bottleneck layer to model global associations, and restores spatial resolution through a convolutional decoder [25]. Swin UNETR, proposed by Hatamizadeh et al., uses a Swin Transformer as the encoder to process multimodal three-dimensional MRI directly and combines it with skip connections and a convolutional decoder, achieving strong performance on the BraTS segmentation task [26]. In addition, taking into account that certain MRI modalities may be unavailable in clinical practice, Zhang et al. proposed mmFormer, which establishes inter-modality feature associations through modality-specific encoders and a cross-modal Transformer, allowing the model to retain a degree of segmentation capability when only a subset of modalities is available [27]. Taken together, these studies indicate that combining the local feature extraction capability of convolution with the long-range modeling capability of the Transformer constitutes one of the principal directions in current brain tumor segmentation research.

Nevertheless, medical image segmentation requires not only the modeling of global information but also the integration of spatial detail and high-level semantics across features at different scales. Feng et al. proposed the cross-scale feature interaction network CSFINet, which aggregates feature patches from different scales through a Patch-wise Interaction Unit (PIU) and promotes information exchange between features at adjacent scales during upsampling through a Feature-wise Interaction Unit (FIU) [1]. The present study adopts this architecture as its basis and applies it to the four-modality brain tumor segmentation task of BraTS 2020.

#### 1.4.3. The Brain Tumor Segmentation Task and the BraTS Dataset

The Brain Tumor Segmentation Challenge (BraTS) is a major public benchmarking platform for multimodal brain tumor magnetic resonance image analysis. Its data comprise multimodal MRI scans that have undergone spatial preprocessing including image co-registration, resampling to a uniform voxel size, and skull stripping; expert-reviewed tumor region annotations; and, for a subset of patients, clinical data such as age, resection status, and survival in days [14,15,28,29,30].

The BraTS challenge has been held continuously since 2012, with the scale of its data and the design of its tasks adjusted from year to year. The RSNA–ASNR–MICCAI BraTS 2021 benchmark compiled by Baid et al. substantially expanded the number of training and validation cases to several thousand and introduced an additional radiogenomics classification task, namely the prediction of MGMT promoter methylation status from imaging. This indicates that the BraTS series of datasets has gradually evolved from a purely segmentation-oriented evaluation platform into an integrated analysis platform encompassing molecular pathology and clinical outcomes [31]. This trend also suggests that tumor segmentation results have research value as an intermediate output for subsequent prognostic analysis.

With regard to segmentation methods, the winning solutions of recent BraTS challenges have largely been built upon adaptations of nnU-Net. In BraTS 2021, Luu and Park obtained further improvements over the existing nnU-Net pipeline by introducing group normalization, an axial attention decoder, and a larger network width [32], indicating that data preprocessing, postprocessing, and training strategies exert a substantial influence on the final performance of brain tumor segmentation.

Regarding data acquisition, this study conducts its experiments on the BraTS 2020 dataset; the copy of the data actually used was obtained from the public Kaggle dataset “Brain Tumor Segmentation (BraTS2020)” [33].

The cohort comprises 235 patients, which were divided according to the study design into 188 training cases (80%) and 47 validation cases (20%). Each patient has four MRI sequences—T1, T1ce, T2, and FLAIR—that have undergone spatial alignment and skull stripping. Segmentation is performed on two-dimensional axial slices, each with a spatial size of 240×240 pixels. In addition, the data include the age, surgical resection status, and survival in days for a subset of patients, which serve as the training and test data for the subsequent overall survival prediction model.

The BraTS 2020 segmentation annotations are stored as a single multi-class segmentation mask, in which each voxel is assigned exactly one label value. The original segmentation labels comprise four classes: (1) background; (2) necrotic and non-enhancing tumor core (NCR/NET); (3) peritumoral edema (ED); and (4) enhancing tumor (ET) [14,15]. The original annotations therefore consist of one background class and three tumor tissue classes.

BraTS segmentation evaluation typically combines the above tumor tissue labels into a single evaluation region: the whole tumor (WT), which comprises NCR/NET, ED, and ET [14,15].

The performance evaluation in this study focuses on the whole tumor region (WT). Accordingly, only at the evaluation stage following inference are NCR/NET, ED, and ET merged—in both the predictions and the ground-truth annotations—into a single foreground class, with the background set to 0, so as to construct a binary WT mask. Dice, precision, and recall are all computed on this binary WT mask.

#### 1.4.4. Overall Survival Prediction and Analysis of Clinical Indicators

In glioma research, overall survival (OS) is one of the most commonly used clinical outcome measures. This study uses the survival information recorded in days in the BraTS dataset, referred to hereafter as survival days, as the regression target of the model. The dataset additionally provides clinical information such as patient age and surgical resection status, which may serve as candidate input features for the survival prediction model [15].

Patient survival, however, may be associated with a variety of factors, including tumor imaging appearance, physiological condition, treatment regimen, and molecular pathological characteristics; relying on tumor imaging or segmentation results alone is therefore generally insufficient to account for prognostic differences among patients. Accordingly, in addition to the tumor segmentation mask, this study incorporates patient age and surgical resection status as candidate clinical features in order to investigate the auxiliary value of different types of clinical information for survival prediction.

Existing clinical research indicates a statistical association between age and overall survival in patients with glioblastoma. In a multivariate survival analysis of glioblastoma patients, Lutterbach et al. found that older age was associated with a higher risk of death and shorter overall survival [5]. This study therefore adopts patient age as a candidate clinical feature, so as to provide physiological background information beyond that available from imaging, and evaluates its effect on survival prediction through ablation experiments.

Huang et al. proposed a hybrid pipeline for glioma overall survival prediction that combines automated tumor segmentation, radiomics, deep features, and clinical variables [34]. Their NLSE-VNet incorporated squeeze-and-excitation and non-local modules to segment tumor subregions from multimodal MRI. Features extracted from the predicted tumor masks—including 2,500 radiomic features, 512 CNN features, age, and tumor grade—were reduced by factor analysis and regressed onto survival days using a random forest model. The average Dice score for the brain tumor segmentation task reached 79%, and the average RMSE for the survival prediction task was as low as 311.5. On an internally split BraTS 2020 cohort, their approach achieved an MAE of 240.05 days and an RMSE of 316.31 days; using ground-truth masks improved the MAE to 225.59 days, highlighting the impact of segmentation quality on downstream prognosis prediction.

Huang et al. incorporated patient age and tumor grade (LGG and HGG, low- and high-grade glioma), in addition to radiomic and deep imaging features, for overall survival regression. Since tumor grade is a clinically derived pathological variable strongly associated with glioma aggressiveness and prognosis, the reported performance should be interpreted as that of a multimodal imaging-plus-clinical model rather than an MRI-only survival prediction model. The experiments reported in the present study lead to the same conclusion.

Beyond age, this study also adopts the patient’s surgical resection status as a candidate clinical feature. Once categorically encoded, resection status can be fused with the tumor mask features, allowing the model to learn the statistical association between the different input variables and survival. In terms of clinical evidence, patient age, tumor grade, and molecular pathological characteristics are established determinants of prognosis in diffuse glioma [3], and maximal safe resection is the surgical objective supported by the available evidence: Molinaro et al. reported that maximal resection of both contrast-enhanced and non-contrast-enhanced tumor is associated with longer survival, with the strength of the association differing across molecular subgroups [8]. On behalf of the RANO resect group, Karschnia et al. further proposed a classification system for extent of resection based on residual tumor volume and validated the association between the resulting resection categories and overall survival in multicenter data, finding that a greater extent of resection was generally associated with a more favorable prognosis [9]. This literature supports the rationale for including resection status among the candidate clinical features. It should be noted, however, that the resection status provided in BraTS 2020 distinguishes only three categories—GTR, STR, and not otherwise specified—and provides no quantitative information such as residual tumor volume, so that the prognostic information it can convey is comparatively limited.

With respect to imaging-based survival prediction methods, Wijethilake et al. conducted a systematic review of studies on glioma survival analysis, grouping existing approaches into those based on radiomic features, those based on deep learning features, and multimodal fusion methods that incorporate clinical and molecular information. They noted that most studies are constrained by limited sample sizes, inconsistent dataset annotations, and a lack of external validation, which makes the generalizability of the resulting models difficult to establish [35]. This observation is consistent with the experimental conditions of the present study and indicates that the ablation results reported here should be regarded as a preliminary comparison obtained on one particular dataset.

In light of the above considerations, this study uses the spatial and morphological features provided by the three-dimensional tumor mask as the principal imaging information and additionally incorporates age and surgical resection status, yielding four input configurations: image only, image combined with age, image combined with resection status, and image combined with both age and resection status. The survival prediction results of these configurations are compared through ablation experiments in order to assess the auxiliary value contributed by the different clinical features.

#### 1.4.5. Explainable Artificial Intelligence in Medical Imaging

Although deep learning models have demonstrated strong performance in brain tumor image segmentation, their highly non-linear computational mechanisms mean that they are often regarded as black-box models, making it difficult for users to trace the image features on which a model bases its segmentation. If a model relies excessively on background noise or on image regions unrelated to the tumor during feature extraction, its decision basis may lack plausibility even when the resulting segmentation is reasonably accurate. Employing feature attribution methods to analyze the influence of different input features on the model output therefore helps in examining whether the model’s segmentation decisions are grounded principally in the tumor lesion and its surrounding regions [36].

Several review articles have recently surveyed the application of explainability methods to medical image analysis. van der Velden et al. classified xAI methods in the medical imaging domain along dimensions such as the scope of the explanation (local or global), model dependence (model-specific or model-agnostic), and the form of the explanation (e.g., heatmaps, examples, or concepts), and observed that attribution methods presented as visual heatmaps are currently the most widely used [37]. Subsequent work has examined the reliability of these attribution methods directly. Adebayo et al. showed that several widely used saliency methods are largely unchanged when the trained model parameters are randomized, so that their output may reflect the input image rather than the model under study [38], and Arun et al. found that saliency maps localize abnormalities unreliably in medical imaging [39]. Attribution results are therefore better regarded as an auxiliary inspection tool than as direct evidence of model correctness. In view of these considerations, the attribution analysis in this study is used solely to observe the image locations on which the model relies when performing tumor segmentation.

This study adopts the SHAP (SHapley Additive exPlanations) framework for its model explainability analysis. SHAP is grounded theoretically in the Shapley value from cooperative game theory and is used to estimate the marginal contribution of each input feature to the model output relative to a reference baseline [36]. In the four-modality MRI segmentation task considered here, the signal at each pixel location in each image channel may influence the model’s tumor segmentation output; attribution values can therefore be used to observe the influence of different modalities and image locations on a specified model output.

For high-dimensional MRI data, this study employs Gradient SHAP to produce pixel-level feature attribution heatmaps. Gradient SHAP builds on Expected Gradients and estimates the contribution of each input feature to the model output using multiple reference samples together with input perturbations [40]. In the signed attribution results, a positive value indicates that the image signal at that location increases the specified tumor-related output relative to the reference baseline, a negative value indicates that it decreases that output, and values close to zero indicate a negligible influence. It should be noted that the sign of an attribution value denotes the direction of influence on the specified output and does not in itself indicate whether the model’s judgment is correct.

## 2. Materials and Methods

This section describes the data sources, preprocessing pipeline, and functional modules of the CSFINet model. The proposed pipeline improves brain tumor segmentation accuracy through a cross-scale feature interaction mechanism and fuses imaging features derived from segmented tumor slices with clinical data for survival prediction.

### 2.1. Data Specification and Experimental Design

**Figure 1:**
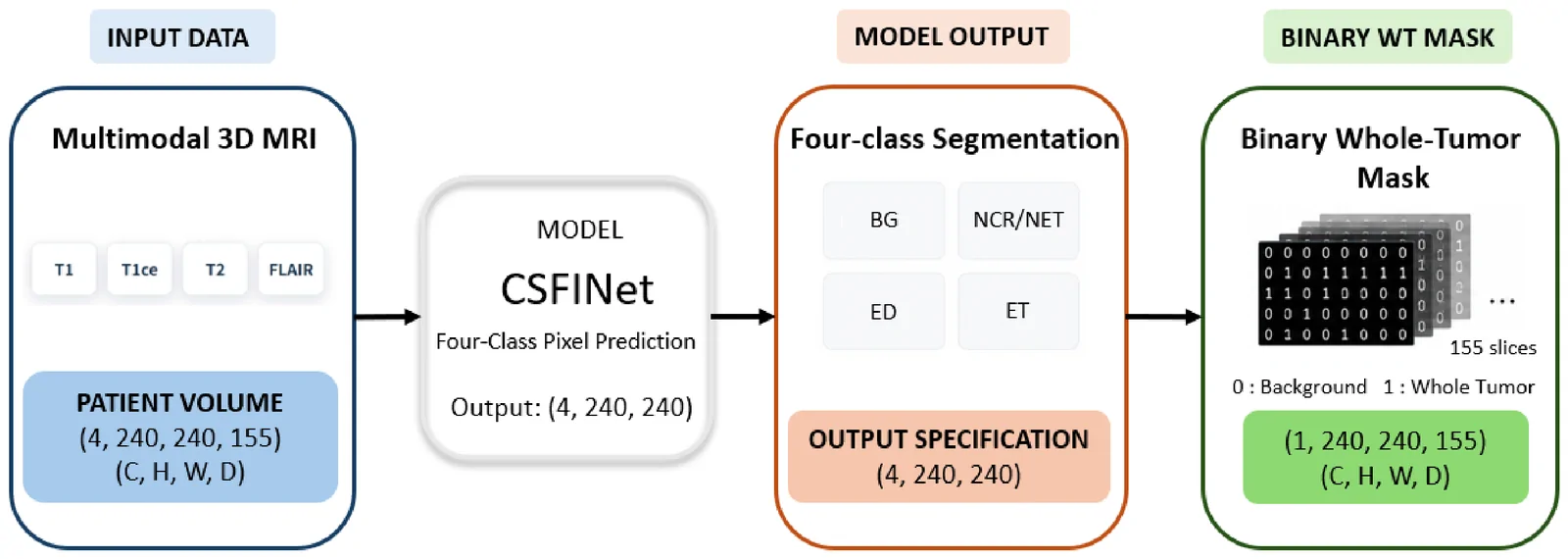
Stage one—schematic of the input and output of the brain tumor image segmentation task.

The first stage of our approach addresses brain tumor image segmentation. Its input consists of the multimodal three-dimensional magnetic resonance images of each patient, comprising the four modalities T1, T1ce, T2, and FLAIR, with a data specification of (4,240,240,155), denoting the number of channels, the height, the width, and the slice depth, respectively. Operating on axial slices, the model takes each image of size (4,240,240) as input to CSFINet and performs a four-class prediction for every pixel: background, necrotic and non-enhancing tumor core (NCR/NET), peritumoral edema (ED), and enhancing tumor (ET). After segmentation, the three tumor classes (NCR/NET, ED, and ET) are merged into a single foreground: the background is retained as 0 and the tumor region is set to 1, yielding a three-dimensional binary whole-tumor mask of size (1,240,240,155) that serves as the image input to the subsequent survival prediction model. The input, model processing, and output of the first stage are illustrated in Figure 1.

**Figure 2:**
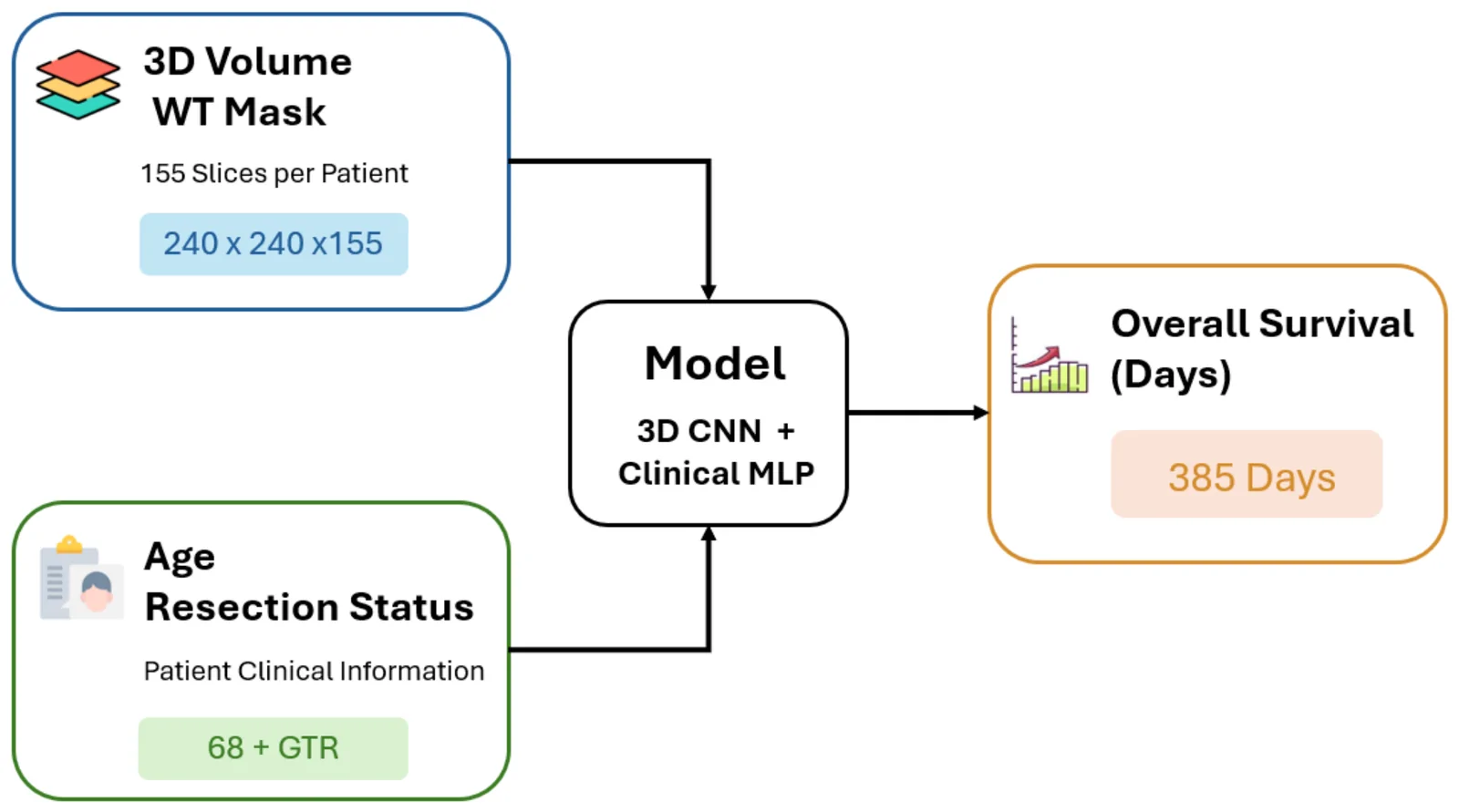
Stage two—schematic of the input and output of the survival prediction task.

The second stage of our approach addresses the prediction of patient survival. The model input comprises the three-dimensional binary whole-tumor mask obtained in the first stage, together with clinical information on the patient’s age and tumor resection status. The original whole-tumor mask of each patient consists of 155 binary images of size 240×240, giving a volume specification of (240,240,155); before being fed to the model, each slice is resized to 128×128, forming three-dimensional image data of size (128,128,155). The model employs a three-dimensional convolutional neural network (3D CNN) to extract the spatial and volumetric features of the tumor mask, and a multilayer perceptron (MLP) to process the standardized age and the one-hot encoded tumor resection status. Finally, the imaging and clinical features are concatenated and fused, and the model outputs a predicted value that serves as the patient’s predicted survival in days. The data input, feature fusion, and output of the second stage are illustrated in Figure 2.

#### 2.1.1. Image Specification and Spatial Dimensions

This study uses the multimodal brain MRI dataset provided by the BraTS 2020 challenge. Each patient has four MRI modalities—T1, T1ce, T2, and FLAIR—and, for a subset of patients, clinical data on age, tumor resection status, and survival in days.

The BraTS 2020 images used in this study have already undergone spatial preprocessing, including co-registration, resampling to a uniform voxel size, and skull stripping. Following co-registration, the four MRI modalities of a given patient lie in the same spatial coordinate system and voxel grid, so that the same voxel position corresponds to the same anatomical location and the four modalities can be stacked along the channel dimension.

To reduce differences in intensity distribution across patients and across MRI modalities, Z-score intensity normalization is additionally applied to each MRI modality of each patient; the method is described in detail in Section 2.3.

The raw images are stored as three-dimensional volume tensors with a spatial resolution of 240×240×155 voxels per modality, preserving anatomical continuity and spatial integrity. The four MRI modalities—T1, T1ce, T2, and FLAIR—are stacked along the channel dimension to form a four-channel input tensor, providing the model with richer multidimensional information.

Due to hardware and efficiency constraints, the three-dimensional volumes are sliced along the axial direction into two-dimensional images, yielding a final input dimensionality of (4,240,240).

The spatial size of each MRI volume is 240×240×155; stacking the four modalities yields a volume tensor of 4×240×240×155. After slicing along the axial direction, each two-dimensional training sample has a size of 4×240×240.

#### 2.1.2. Survival Labels and Censoring

BraTS 2020 distributes a single scalar field, Survival_days, for each subject with clinical data. Neither an event indicator nor a date of last follow-up is provided, and the official challenge protocol treats this value as an observed overall survival time measured from the date of the preoperative scan, evaluating predictions by classification into short (<10 months), mid (10–15 months), and long (>15 months) survivors of patients who underwent gross total resection [14,15]. All 235 survival times used in this study are therefore treated as complete, uncensored observations, because no censoring information exists in the released data.

#### 2.1.3. Patient-Level Data Split and Sample Size

The 235 BraTS 2020 subjects with survival labels were partitioned at the *patient* level into 188 training and 47 test subjects. The unit of randomization is the subject: all 155 axial slices and all four modalities of a given subject are assigned to exactly one partition. The partition was produced by a single simple random draw without stratification, performed once with the fixed global seed 42 before any model was trained, and was not changed thereafter. The same partition is used for the segmentation stage, for the survival stage, and for all four ablation configurations, so that the comparisons reported in Section 3.2 and Section 3.3.2 refer to the same patients. The identifiers of the 188 training and 47 test subjects are listed in Table S1 of the Supplementary Materials Section S1 so that the split can be reproduced exactly.

No image-level (slice-level) partitioning is used at any stage. A slice-level split would offer a larger and more evenly balanced training set and more stable per-slice metrics, but at the decisive cost of information leakage and an optimistically biased estimate of performance. Within a subject, all slices are used and each slice carries the four-class label map; no subject and no slice crosses the training/test boundary at any point in either the segmentation or the survival experiments.

Regarding the split ratio, “according to Vrigazova (2021) and Tan et al. (2021), the most common train–test splits in the literature are 70:30 and 80:20, offering a good balance by providing enough data for both training and testing, and are often selected for their robustness and reliability in various contexts” [41,42,43]; the 80:20 ratio was adopted for this reason. The sample size itself was determined by the dataset rather than chosen: BraTS 2020 provides survival labels for 235 subjects, and all 235 were used, so no larger sample was available within this cohort.

The distribution of resection status in the two partitions is shown in Table 1. The split was not stratified by resection status or by survival, so the balance achieved is that of the random draw.

### 2.2. Core Network Architecture

The first stage of this study adopts CSFINet as the core network for brain tumor image segmentation. The model input is a four-channel two-dimensional MRI slice composed of FLAIR, T1, T1ce, and T2, with a tensor size of 4×240×240. The model extracts image features at different scales through an encoder, and progressively restores the spatial resolution through cross-scale feature interaction, feature alignment, and a decoder. The final layer of the model outputs four class scores (logits) for each pixel, with an output size of 4×240×240, in which the four output channels correspond, in order, to background, NCR/NET, ED, and ET. After the softmax function, each pixel is assigned a predicted likelihood for each of the four classes.

Whereas conventional U-Net relies chiefly on skip connections to concatenate shallow- and deep-level features directly, CSFINet additionally introduces cross-scale feature interaction and feature alignment mechanisms, enabling the model to integrate deep semantic information while preserving local spatial detail. This design addresses the indistinct boundaries, irregular shapes, and large scale variation commonly encountered in brain tumor images, improving the accuracy and stability of the segmentation results.

#### 2.2.1. CSFINet-Based Image Segmentation Architecture

**Figure 3:**
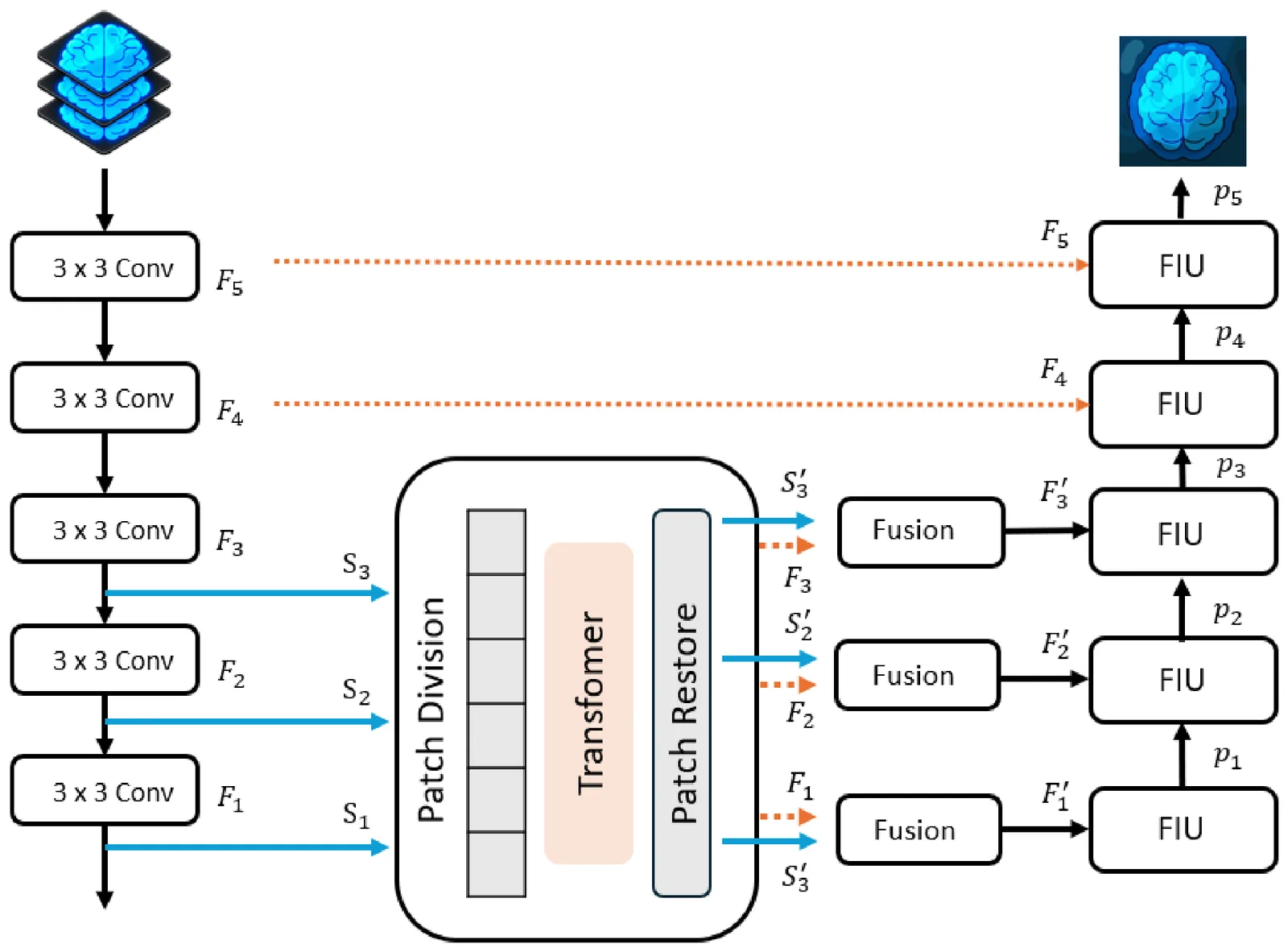
Network architecture of CSFINet. Black solid arrows denote the main forward path: the convolutional encoder produces the multi-scale features $F_{1}$–$F_{5}$, the fused features $F_{1}^{\prime }$–$F_{3}^{\prime }$ are passed to the Feature-wise Interaction Units (FIU), and the FIU outputs $p_{1}$–$p_{5}$ are propagated upward to the final segmentation. Blue solid arrows denote the cross-scale path through the Patch-wise Interaction Unit: the encoder features $S_{1}$–$S_{3}$ enter Patch Division, and the Transformer-refined features $S_{1}^{\prime }$–$S_{3}^{\prime }$ leave Patch Restore and enter the Fusion modules. Orange dashed arrows denote skip connections that pass the encoder features $F_{1}$–$F_{5}$ directly to the Fusion modules and the FIUs. This schematic diagram was redrawn based on the content presented in [1].

As shown in Figure 3, the overall CSFINet architecture consists mainly of a convolutional feature encoder, a Patch-wise Interaction Unit (PIU), a feature fusion module, and a Feature-wise Interaction Unit (FIU); its processing flow comprises multi-scale feature encoding, cross-scale feature interaction, feature fusion, and restoration of the spatial resolution.

The model first feeds the multimodal MRI images into the convolutional feature encoder to extract image features at different resolutions. As shown on the left side of Figure 3, the encoder progressively reduces the spatial resolution of the feature maps through convolution and max pooling operations, producing feature representations at different scales. The shallow-level features preserve local details such as tumor boundaries, texture, and spatial position, whereas the deep-level features contain higher-order semantic information that helps to characterize the overall structure of the tumor region.

To strengthen information transfer among features at different scales, CSFINet introduces a Patch-wise Interaction Unit (PIU) in the encoding stage. The PIU converts feature maps at different scales into patch tokens and employs a Transformer to establish associations among cross-scale features, allowing shallow-level spatial detail and deep-level semantic information to interact. Through this mechanism, the model can draw on local features and global information simultaneously, reducing the loss of fine lesion information that conventional convolutional neural networks may incur during successive downsampling, and thereby improving its ability to identify irregular tumor shapes and indistinct boundaries.

The cross-scale features produced by the PIU are then fused with the corresponding scale features of the encoder. As shown by the Fusion module in Figure 3, the two sets of features are first concatenated and then integrated across channels by a 1×1 convolution, forming the fused features required for the subsequent feature restoration stage.

In the feature restoration stage, CSFINet uses the Feature-wise Interaction Unit (FIU) to integrate progressively the feature information of adjacent scales. The FIU upsamples the low-resolution deep features and concatenates them with the fused features at the corresponding scale; in addition, the module uses an estimated displacement field to align features of different scales spatially, so as to reduce the semantic gap and spatial offset that may arise during feature fusion. Through layer-by-layer feature interaction, alignment, and resolution restoration, the model ultimately produces a pixel-level brain tumor segmentation result.

In summary, the first-stage image segmentation model of this study exploits the PIU and FIU of CSFINet to strengthen cross-scale feature interaction and spatial alignment, enabling the model to cope with the large scale variation, indistinct boundaries, and complex morphological variation encountered in brain tumor images. The resulting tumor segmentation mask serves as the image input to the subsequent survival prediction stage.

#### 2.2.2. Baseline Model Comparison

To determine whether the cross-scale feature interaction and feature alignment mechanisms of CSFINet improve upon the performance of conventional convolutional segmentation models, a basic U-Net was additionally implemented as a baseline. U-Net adopts a symmetric encoder–decoder architecture in which the encoder progressively extracts high-level semantic features from the image through convolution and max pooling, while the decoder progressively restores the spatial resolution through upsampling and uses skip connections to concatenate encoder and decoder features at the same scale, thereby preserving boundary information and spatial localization capability. To ensure a fair comparison, U-Net and CSFINet use the same four-modality MRI input, the same training and test patient data, the same image normalization method, loss function, learning rate, batch size, random seed, early stopping criterion, and the same inference and postprocessing pipeline. The only difference between the two models lies in the design of the network architecture; holding all other experimental conditions constant therefore allows the actual influence of the cross-scale feature interaction and feature alignment mechanisms proposed in CSFINet on brain tumor segmentation performance to be assessed.

### 2.3. Image Preprocessing Pipeline

This study uses the BraTS 2020 MRI data, in which each patient has four MRI modalities: FLAIR, T1, T1ce, and T2. To reduce differences in intensity distribution across imaging modalities and across patients, intensity normalization is applied to each MRI modality before model training, so that the image intensity distributions have a more consistent scale and training stability is improved.

#### Z-Score Intensity Normalization

Prior to training the segmentation model, Z-score normalization is applied separately to each MRI modality of each patient, computed as follows:

(1) $$I_{\mathrm{norm}}=\frac{I-\mu }{\sigma +\epsilon }$$

**Figure 4:**
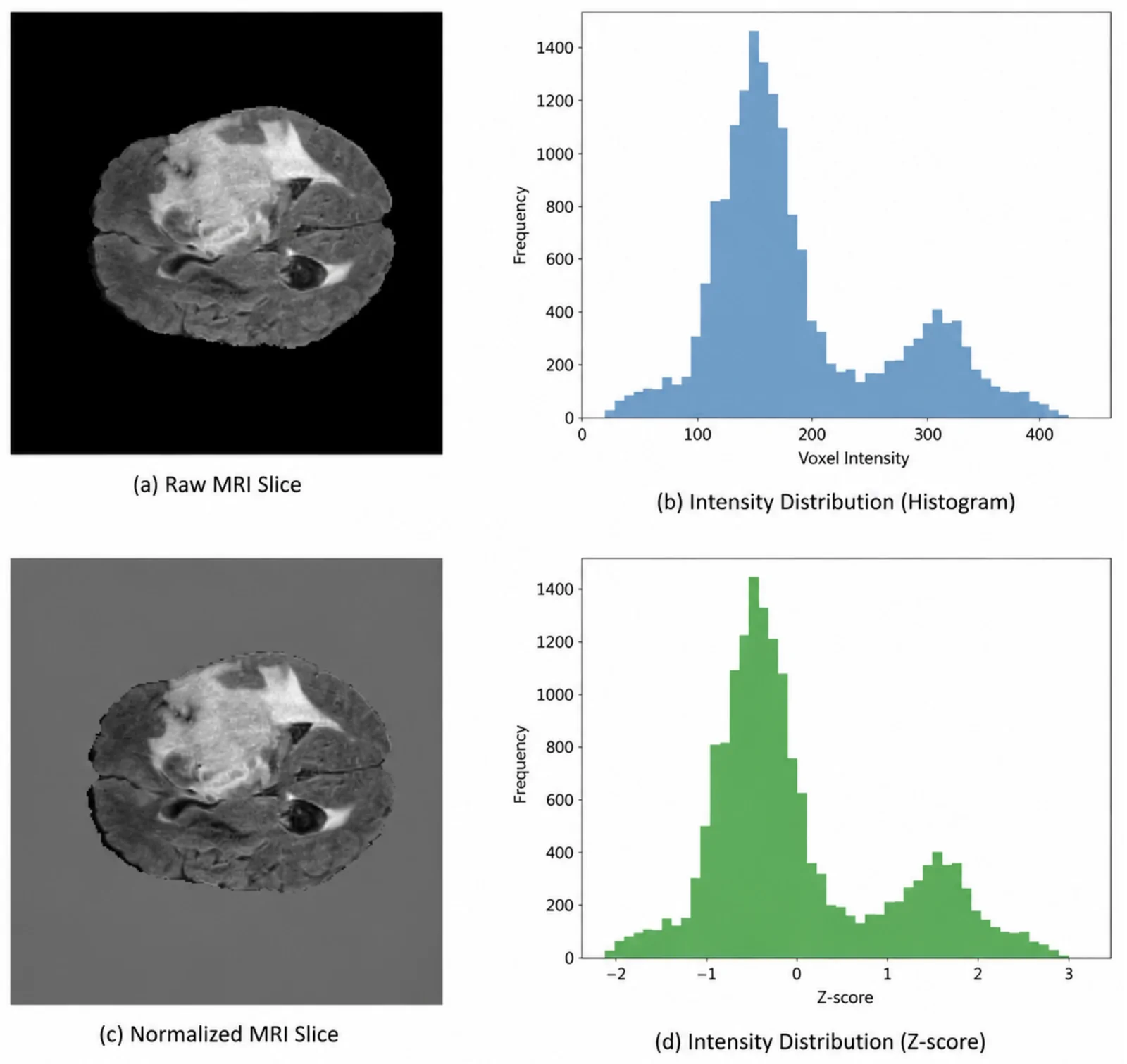
Comparison of slice images and histograms before and after MRI intensity normalization.

Here, *I* denotes the original MRI intensity value, μ the mean intensity of the image volume, σ the standard deviation of the image volume, and ϵ a small constant introduced to avoid division by zero when the standard deviation vanishes. In this study, $\epsilon =1\times 10^{-8}$. After normalization, the intensities of each MRI modality follow a distribution with a mean close to 0 and a standard deviation close to 1, allowing the model to learn tumor features across modalities more stably during training. An illustrative example is presented below to demonstrate the procedure, as shown in Figure 4.

### 2.4. Clinical Data Preprocessing Pipeline

In the survival prediction stage, patient age and extent of resection are used as auxiliary clinical features. Since the clinical data comprise both continuous values and categorical variables, numerical standardization and categorical encoding are applied separately before the data are fed to the model, facilitating feature fusion and training.

#### 2.4.1. Age Standardization

To prevent age from dominating the prediction and to reduce scale disparity among input features, patient age was standardized using the Z-score, formulated as:

(2) $$Age_{\mathrm{norm}}=\frac{Age-\mu_{Age,\mathrm{train}}}{\sigma_{Age,\mathrm{train}}+\epsilon }$$

Here, Age denotes the patient’s original age, and $\mu_{Age,\mathrm{train}}$ and $\sigma_{Age,\mathrm{train}}$ denote the mean and standard deviation of age in the training set, respectively. In this study of the 188 training records, the mean age is 60.9869 years and the standard deviation is 12.1188 years.

Once standardized, this age value is fed, together with the one-hot encoded vector of the tumor resection status, into the clinical feature processing network, and is then fused with the imaging features to predict the patient’s survival.

#### 2.4.2. Encoding of Resection Status

Tumor resection status is a categorical clinical feature and cannot be fed to a neural network directly in textual form. This study therefore uses one-hot encoding to convert resection status into a numerical vector. The resection statuses present in the data are gross total resection (GTR), subtotal resection (STR), and unknown or unlabeled (NA), encoded as follows:

(3) $$X_{\mathrm{status}}=\left\{\begin{array}{cc} {}[1,0,0], & \mathrm{if}\;\mathrm{the}\;\mathrm{resection}\;\mathrm{status}\;\mathrm{is}\;\mathrm{GTR}\\ {}[0,1,0], & \mathrm{if}\;\mathrm{the}\;\mathrm{resection}\;\mathrm{status}\;\mathrm{is}\;\mathrm{STR}\\ {}[0,0,1], & \mathrm{if}\;\mathrm{the}\;\mathrm{resection}\;\mathrm{status}\;\mathrm{is}\;\mathrm{NA} \end{array}\right.$$

Encoding NA as a third level of the one-hot vector is an instance of the *missing-indicator method*, and it is named as such here rather than being presented as a genuine clinical category. The missing-indicator method is known to yield biased estimates when the objective is to estimate the effect of an exposure [44], but it is defensible in a prediction model provided that the same missingness mechanism is expected to operate at deployment and that no causal interpretation of the corresponding model parameter is attempted [45]. Because BraTS distributes the label as “NA” without any information on why the status is absent, the missingness mechanism cannot be verified. Multiple imputation cannot be justified either: with a single categorical predictor and no auxiliary variables that are predictive of resection status, an imputation model would add assumptions without adding information. In addition, the interpretive limitation of the NA category is stated explicitly in Section 4.2.

### 2.5. Survival Prediction Model and Ablation Study Design

The second stage of this study uses the binary whole-tumor mask as the image input to the survival prediction model. During training, the ground-truth segmentation annotations provided by the BraTS 2020 dataset are used, with NCR/NET, ED, and ET merged into a binary whole-tumor mask; during validation, the binary predicted mask produced by CSFINet in the first stage is used. Both types of mask consist of 155 consecutive slices and characterize the spatial distribution and morphology of the tumor in three dimensions. Note that the survival models are *trained on expert ground-truth masks* but *tested on CSFINet-predicted masks*. Training on expert annotations avoids compounding segmentation error with label noise during optimization, while evaluating on predicted masks reproduces the deployment condition, in which no expert annotation is available. The consequence is that the reported survival errors carry the segmentation error of the first stage, and they are not the errors that would be obtained if ground-truth masks were available at inference time.

In addition to the tumor mask, patient age and tumor resection status are adopted as candidate clinical features, giving rise to four survival prediction models: image only, image combined with age, image combined with resection status, and image combined with both age and resection status. The term “image” as used here refers to the binary whole-tumor mask rather than to the original multimodal MRI. The four models share the same imaging feature extraction and differ only in whether age and resection status are included, so as to compare the auxiliary value of the different clinical features for survival prediction.

Four considerations motivate the use of a binary whole-tumor mask rather than the three-class subregion map as the image input of the second stage. First, the whole-tumor region is the most reliably delineated of the BraTS regions in the published literature, in which whole-tumor Dice consistently exceeds tumor-core and enhancing-tumor Dice by a considerable margin (Table 2, which will be detailed in Section 3.2); because the second stage consumes the output of the first, using the most reliably segmented region limits the propagation of segmentation error into the survival model. Second, the quantity encoded by the 3D CNN is essentially the total lesion burden together with its three-dimensional shape and slice-wise distribution, which is the imaging descriptor most consistently reported as prognostic. Third, with 188 training patients, a three-channel subregion input triples the input dimensionality of the encoder and increases the risk of overfitting. Fourth, merging the classes into a binary mask makes the training input (the ground-truth annotation) and the inference input (the CSFINet prediction) structurally identical. The corresponding limitation—that enhancing-tumor and necrotic-core volumes carry prognostic information which a binary mask discards—is stated in Section 4.2 and is listed in Future Work.

#### 2.5.1. Imaging Feature Extraction

The model takes the binary whole-tumor mask as its image input. The training stage uses the BraTS ground-truth binary mask, and the validation stage uses the binary mask predicted by CSFINet. The mask of each patient consists of 155 consecutive slices with an original size of 240×240×155. To reduce the computational load and to conform to the model’s input requirements, each slice is resized to 128×128, so that the input image tensor $X_{\mathrm{img}}$ can be expressed as:

(4) $$X_{\mathrm{img}}\in R^{1\times 128\times 128\times 155}.$$

Here, 1 denotes the single-channel tumor mask, 155 the number of MRI slices per patient, and 128×128 the resized image dimensions. Imaging feature extraction employs a 3D CNN encoder to learn the spatial features of the tumor mask. Through three-dimensional convolution, the model captures the spatial distribution, volumetric variation, and morphological characteristics of the tumor across slices. The imaging feature vector $F_{\mathrm{img}}$ output by the image encoder can be expressed as:

(5) $$F_{\mathrm{img}}\in R^{1\times 128}.$$

Here, 1 denotes the single-channel tumor mask, 155 the number of MRI slices per patient, and 128×128 the resized image dimensions.

Imaging feature extraction employs a 3D-CNN encoder to transform the input tensor $X_{\mathrm{img}}$ into the imaging feature vector $F_{\mathrm{img}}$. Through three-dimensional convolution, the model captures the spatial distribution, volumetric variation, and morphological characteristics of the tumor across slices. The relationship between the input tensor and the output feature vector can be expressed as:

(6) $$F_{\mathrm{img}}={\mathrm{Encoder}}_{3D\text{-}\mathrm{CNN}}\left(X_{\mathrm{img}}\right)\in R^{1\times 128}.$$

Here, ${\mathrm{Encoder}}_{3D\text{-}\mathrm{CNN}}\left(\cdot \right)$ denotes the feature extraction operation of the 3D-CNN encoder, and $F_{\mathrm{img}}$ denotes the resulting 128-dimensional imaging feature vector. This feature vector characterizes the three-dimensional spatial distribution and morphology of the patient’s tumor region and serves as the source of imaging information for the subsequent feature fusion stage.

#### 2.5.2. Clinical Feature Extraction

Depending on the input configuration of the ablation study, this study uses patient age, tumor resection status, or both clinical features. After Z-score standardization, patient age forms the one-dimensional continuous feature $X_{\mathrm{age}}$; after one-hot encoding, tumor resection status forms the three-dimensional categorical feature $X_{\mathrm{status}}$. When the model uses age and resection status together, $X_{\mathrm{age}}$ and $X_{\mathrm{status}}$ are concatenated along the feature dimension to form the four-dimensional clinical feature $X_{\mathrm{age}+\mathrm{status}}$, that is, $X_{\mathrm{age}+\mathrm{status}}=\left[X_{\mathrm{age}},X_{\mathrm{status}}\right]$. The three clinical inputs can therefore be expressed as:

(7) $$X_{\mathrm{age}}\in R^{1\times 1},\;X_{\mathrm{status}}\in R^{1\times 3},\;X_{\mathrm{age}+\mathrm{status}}=\left[X_{\mathrm{age}},X_{\mathrm{status}}\right]\in R^{1\times 4}.$$

Here, [·,·] denotes concatenation along the feature dimension. In the model that uses age alone, the actual input to the clinical MLP is the Z-score standardized age value rather than the patient’s original age; in the models that use resection status, the input to the clinical MLP is the three-dimensional one-hot vector formed by the classes GTR, STR, and NA.

Since age and resection status are both low-dimensional clinical variables, concatenating the raw clinical features directly with the 128-dimensional imaging features would limit the model’s representational capacity for clinical information to the raw values or categorical encodings. This study therefore designs a clinical multilayer perceptron (Clinical MLP) consisting of two fully connected layers, which first maps the low-dimensional clinical input to a 32-dimensional hidden feature space and then transforms it into a 16-dimensional clinical feature vector. Both fully connected layers are followed by a ReLU non-linear activation function, allowing the model to learn possible non-linear relationships between the clinical variables and survival rather than merely a linear weighting of the raw inputs.

**Figure 5:**
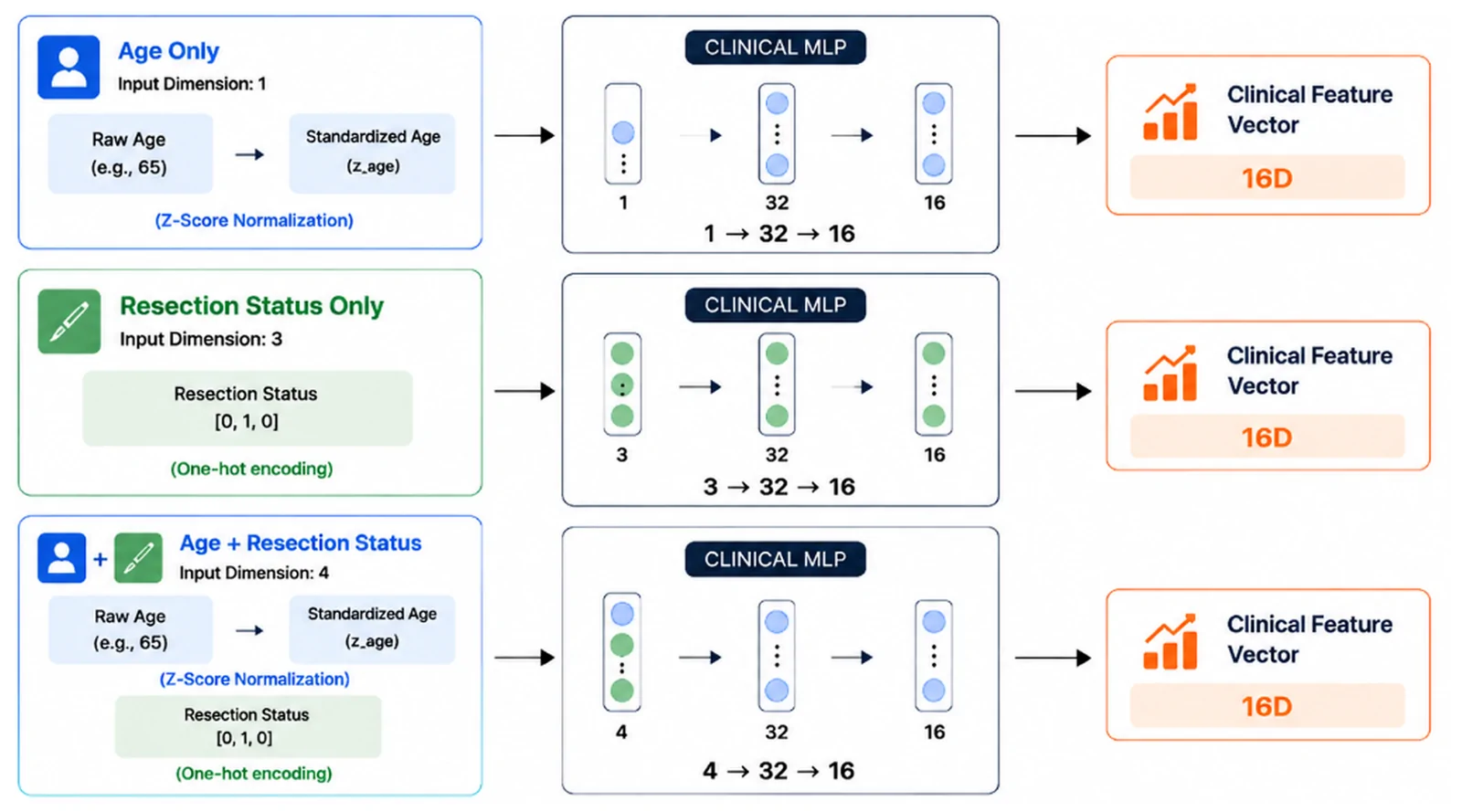
Clinical MLP architectures for the different clinical feature input configurations (Note: This figure was conceived by the author, with OpenAI GPT-5.6 Sol used to assist with revision and layout.).

A Clinical MLP with the corresponding input dimensionality is designed for each clinical input configuration. When age alone is used, the network dimensions are 1→32→16; when resection status alone is used, they are 3→32→16; and when age and resection status are used together, they are 4→32→16. The network architectures for the three clinical inputs are shown in Figure 5.

Although the clinical input dimensionality differs across the three models, each transforms the clinical information into a fixed 16-dimensional clinical feature vector through two fully connected layers. The feature transformation can be expressed as:

(8) $$\begin{array}{cc} H_{\mathrm{cli}}\; & =\mathrm{ReLU}\left(X_{\mathrm{cli}}W_{\mathrm{cli}}^{\left(1\right)}+b_{\mathrm{cli}}^{\left(1\right)}\right)\\ F_{\mathrm{cli}}\; & =\mathrm{ReLU}\left(H_{\mathrm{cli}}W_{\mathrm{cli}}^{\left(2\right)}+b_{\mathrm{cli}}^{\left(2\right)}\right) \end{array}$$

Here, $X_{\mathrm{cli}}$ denotes, according to the model configuration, $X_{\mathrm{age}}$, $X_{\mathrm{status}}$, or $X_{\mathrm{age}+\mathrm{status}}$; $W_{\mathrm{cli}}^{\left(1\right)}$ and $W_{\mathrm{cli}}^{\left(2\right)}$ denote the weight matrices of the two fully connected layers; and $b_{\mathrm{cli}}^{\left(1\right)}$ and $b_{\mathrm{cli}}^{\left(2\right)}$ denote the corresponding bias terms. $H_{\mathrm{cli}}$ is the 32-dimensional hidden feature, and $F_{\mathrm{cli}}$ is the 16-dimensional clinical feature vector finally output by the Clinical MLP.

This architecture constitutes the clinical feature representation adopted for the ablation study. Although the three Clinical MLPs have different input dimensionalities, each outputs a 16-dimensional clinical feature, so that all can subsequently be concatenated with the 128-dimensional imaging features to form a 144-dimensional fused feature. Standardizing the clinical output dimensionality gives the three models that incorporate clinical information a consistent feature fusion interface and downstream prediction architecture, reducing structural differences arising from differing fused-feature dimensionalities and allowing the ablation study to focus more directly on the influence of the different clinical information configurations—age, resection status, and age combined with resection status—on survival prediction. The image-only model uses neither the Clinical MLP nor clinical feature fusion.

#### 2.5.3. Feature Fusion

The three models—image combined with age, image combined with resection status, and image combined with both age and resection status—all concatenate the 128-dimensional imaging feature with the 16-dimensional clinical feature:

(9) $$F_{\mathrm{fusion}}=\left[F_{\mathrm{img}},F_{\mathrm{cli}}\right],$$

where [·,·] denotes the concatenation operation. The dimensionality of the fused feature is therefore:

(10) $$F_{\mathrm{fusion}}\in R^{1\times 144}.$$

Although the three models use different raw clinical features, each clinical branch outputs a 16-dimensional feature, so that the fused feature dimensionality is 144 in every case. The image-only model performs no feature concatenation and instead feeds the 128-dimensional imaging feature directly into the prediction network.

### 2.6. Survival Prediction

For the three models—image combined with age, image combined with resection status, and image combined with both age and resection status—the imaging and clinical features are concatenated into a fused feature vector and fed into a Fusion MLP for survival prediction.

**Figure 6:**
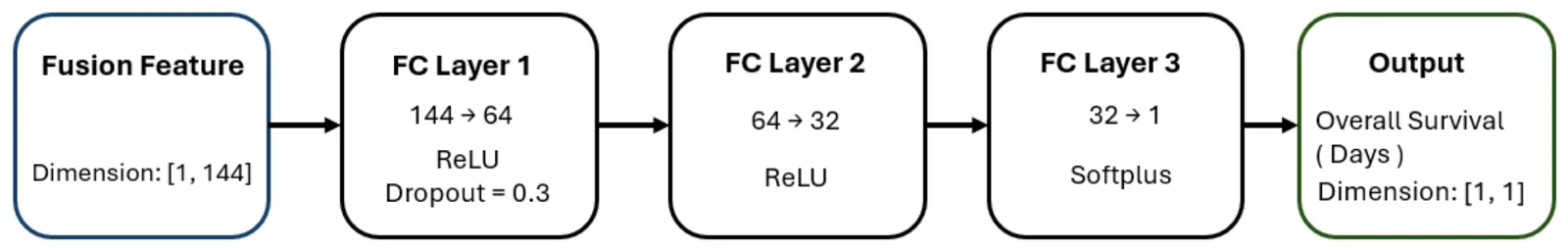
Architecture of the survival prediction model incorporating clinical features.

The Fusion MLP consists of three fully connected (FC) layers. The first layer maps the 144-dimensional fused feature to 64 dimensions, uses ReLU as the non-linear activation function, and applies dropout to reduce the risk of overfitting. The second layer maps the 64-dimensional feature to 32 dimensions, likewise with a ReLU activation. The third layer maps the 32-dimensional feature to a single continuous value representing the predicted survival in days. The overall architecture is shown in Figure 6.

For the models that incorporate clinical features, the 128-dimensional feature produced by the imaging branch and the 16-dimensional feature produced by the clinical branch are concatenated to form the fused feature $F_{\mathrm{fusion}}\in R^{B\times 144}$. where *B* denotes the batch size. Following the PyTorch fully connected layer formulation $Y=XW^{T}+b$, the computation of the Fusion MLP can be expressed as follows:

(11) $$h_{1}={\mathrm{Dropout}}_{p=0.3}\left(\mathrm{ReLU}\left(F_{\mathrm{fusion}}W_{1}^{T}+b_{1}\right)\right)$$

(12) $$h_{2}=\mathrm{ReLU}\left(h_{1}W_{2}^{T}+b_{2}\right)$$

(13) $${\hat{Y}}_{\mathrm{survival}}=\mathrm{Softplus}\left(h_{2}W_{3}^{T}+b_{3}\right)$$

Here, $h_{1}\in R^{B\times 64}$ $h_{2}\in R^{B\times 32}$ and ${\hat{Y}}_{\mathrm{survival}}\in R^{B\times 1}$ The dimensions of the weight matrices and bias vectors of the three fully connected layers are, respectively,

(14) $$\begin{array}{cccc} W_{1} & \in R^{64\times 144}\; & b_{1} & \in R^{64}\\ W_{2} & \in R^{32\times 64}\; & b_{2} & \in R^{32}\\ W_{3} & \in R^{1\times 32}\; & b_{3} & \in R^{1} \end{array}$$

The dimensional transformations at each layer are therefore, in order,

(15) (B×144)(144×64)→B×64,

(16) (B×64)(64×32)→B×32,

(17) (B×32)(32×1)→B×1

The bias vectors are broadcast along the batch dimension and added to the feature vector of every sample in the batch. A dropout layer with a rate of 0.3 follows the first fully connected layer in order to reduce the risk of overfitting, and a Softplus activation follows the final fully connected layer to ensure that the predicted survival is positive.

The image-only model uses neither clinical features nor feature concatenation, and instead feeds the 128-dimensional imaging feature directly into an image-only MLP. Its network dimensions are, in order:

(18) 128→128→64→32→1.

The hidden layers of the image-only model likewise use ReLU activations and dropout, and the output layer likewise uses a Softplus function. Since survival cannot be negative, all four models constrain their outputs to non-negative continuous values by means of Softplus.

### 2.7. Ablation Study Design for the Different Input Feature Configurations

To evaluate the auxiliary value of age and resection status for survival prediction, four input feature configurations were constructed under identical data splits, image inputs, and training settings. Every model uses the binary tumor mask produced by CSFINet as its image input, and includes age and resection status according to the experimental configuration. The four model configurations are listed in Table 3.

Apart from the input feature configuration, all four models use the same data split, image preprocessing, image encoder, number of training epochs, and loss function, ensuring that the ablation comparison is conducted under consistent experimental conditions.

**Figure 7:**
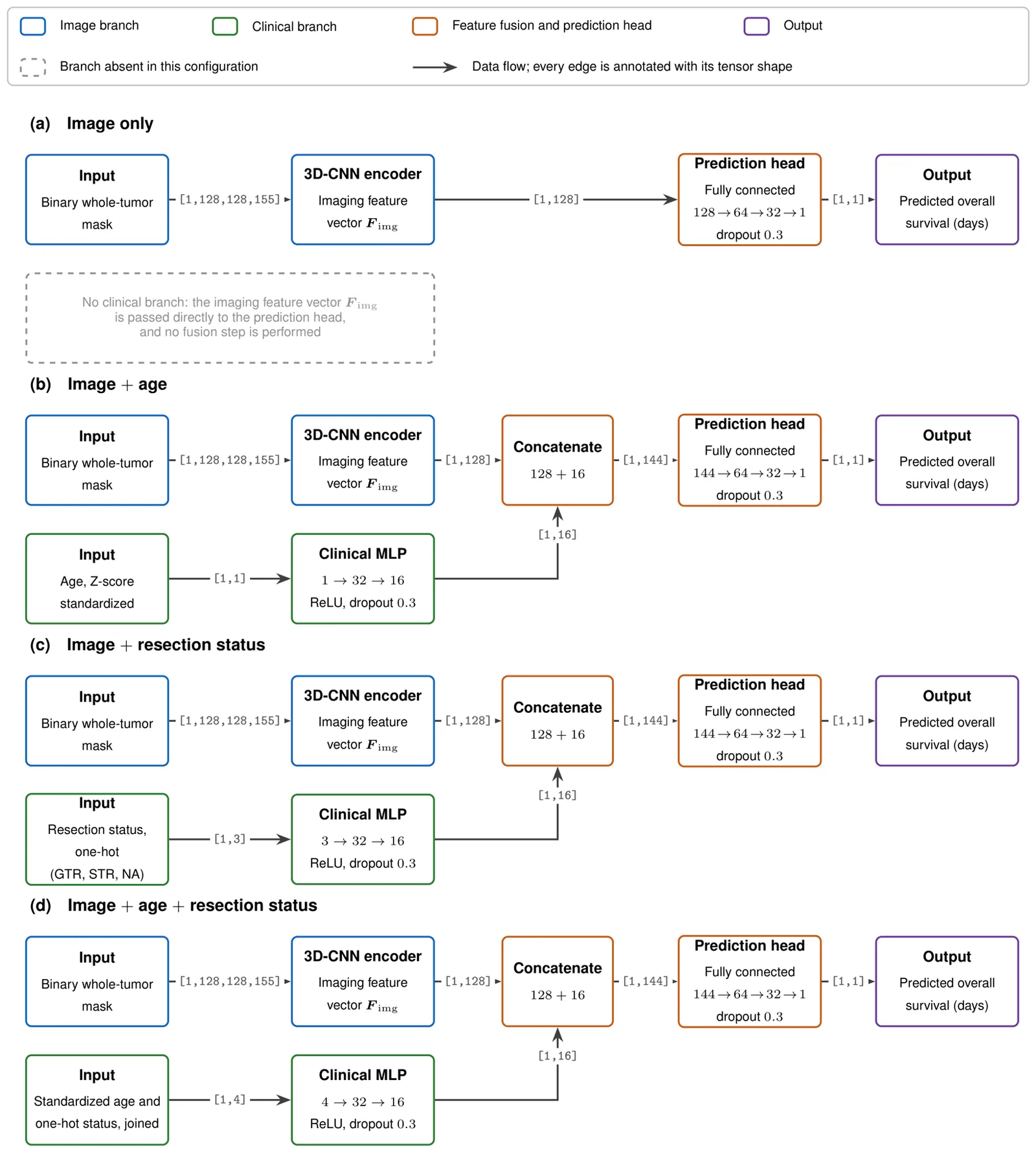
Architectures of the four survival prediction configurations. (**a**) image only; (**b**) image with age; (**c**) image with resection status; (**d**) image with age and resection status. The image branch is identical in all four panels: the binary whole-tumor mask, resampled from 240×240×155 to 128×128×155, is encoded by the 3D CNN into the 128-dimensional vector $F_{\mathrm{img}}$. The panels differ only in the clinical branch, which is absent in (**a**) and is shown there as a dashed outline so that the four rows remain aligned. Every edge is annotated with the shape of the tensor that passes along it.

The architectures of the four configurations are shown in Figure 7. The four configurations differ only in the clinical branch. In every configuration the image branch is identical: the binary whole-tumor mask $X_{\mathrm{img}}\in R^{1\times 128\times 128\times 155}$ is passed through the 3D-CNN encoder to yield the 128-dimensional imaging feature vector $F_{\mathrm{img}}$. (a) In the image-only model, $F_{\mathrm{img}}$ is passed directly to the fully connected head, which outputs a single predicted survival value in days; there is no clinical branch and no fusion step. (b) In the image-with-age model, the Z-score standardized age forms a one-dimensional input to a clinical MLP with dimensions 1→32→16, and the resulting 16-dimensional clinical vector is concatenated with $F_{\mathrm{img}}$ to form a 144-dimensional fused representation before the head. (c) In the image-with-resection-status model, the three-dimensional one-hot vector (GTR, STR, NA) is passed to a clinical MLP with dimensions 3→32→16, and fusion proceeds as in (b). (d) In the image-with-age-and-resection-status model, the standardized age and the one-hot vector are concatenated into a four-dimensional clinical input, which is passed to a clinical MLP with dimensions 4→32→16, and fusion again proceeds as in (b). The fully connected head, the dropout rate of 0.3, the fused feature dimension of 144, the loss function, the optimizer, the number of epochs, and the data split are identical across all four panels.

### 2.8. Training Strategy and Loss Function Design

This study comprises two tasks: brain tumor image segmentation and survival prediction. Since the two tasks differ in input data type and prediction target, a separate training strategy and loss function is designed for each. The segmentation model targets pixel-level classification of the tumor region, whereas the survival prediction model targets a continuous value.

#### 2.8.1. Training Strategy for the Image Segmentation Model

In the segmentation task, multimodal MRI images serve as the input and the tumor annotation mask as the supervisory signal. Since each patient’s MRI volume comprises 155 2D slices, training directly on the complete 3D volume would impose a heavy GPU memory burden; a 2D slice-based training strategy is therefore adopted, decomposing each 3D volume into consecutive 2D slices for training.

During training, the data of one patient are loaded at a time and that patient’s 155 slices are fed to the model in batches. To reduce GPU memory requirements, the patient batch size is set to 1 and the slice batch size to 10, and a gradient accumulation strategy is adopted. The model first computes the segmentation loss for each batch of slices, accumulates the gradients over all slices of the same patient, and then updates the model parameters. This approach preserves the training information of a complete patient volume under limited hardware resources.

During training, the segmentation model performs four-class pixel classification, with the class set defined as:

(19) C={Background,NCR/NET,ED,ET},|C|=4.

Let the four-class logits output by the model for pixel *p* be $z_{p}=\left[z_{p,0},z_{p,1},z_{p,2},z_{p,3}\right]$. After the softmax, the probability that pixel *p* belongs to class *c* is:

(20) $${\hat{y}}_{p,c}=\frac{\exp(z_{p,c})}{\sum_{j=0}^{|C|-1}\exp\left(z_{p,j}\right)}.$$

This study uses the multi-class cross-entropy loss as the segmentation loss function:

(21) $$L_{\mathrm{seg}}=-\frac{1}{N}\sum_{p=1}^{N}\sum_{c=0}^{|C|-1}y_{p,c}\log\left({\hat{y}}_{p,c}\right),$$

where *N* denotes the total number of pixels in a batch, $y_{p,c}$ the one-hot ground-truth class label of pixel *p*, and ${\hat{y}}_{p,c}$ the model’s predicted probability that pixel *p* belongs to class *c*. By minimizing this loss function, the model learns to distinguish among the four classes background, NCR/NET, ED, and ET.

#### 2.8.2. Training Strategy for the Survival Prediction Model

In the survival prediction task, the binary whole-tumor mask volume serves as the common image input. During training, the ground-truth binary tumor masks of the 188 training patients are used; once the model weights have been trained, the binary masks predicted by CSFINet for the 47 validation patients are fed to the model to evaluate survival prediction performance. Depending on the ablation configuration, the model additionally takes patient age and resection status as input, and outputs a single predicted survival value. The four models use the same 188 training patients, 47 validation patients, image size, number of training epochs, loss function, and model weight selection procedure, and differ only in the configuration of clinical input features, so as to ensure a fair ablation comparison.

Since survival data may contain extreme values or exhibit substantial inter-individual variation, using mean squared error alone would render training susceptible to outlying samples. This study therefore adopts the Huber loss, which approximates the squared error for small residuals and the absolute error for large residuals, thereby combining stable training with reduced sensitivity to outliers.

The Huber loss is defined as follows:

(22) $$L_{survival}=\left\{\begin{array}{cc} \frac{1}{2}{\left(y-\hat{y}\right)}^{2}, & \mathrm{if}\;|y-\hat{y}|\leq \delta \\ \delta \left(|y-\hat{y}|-\frac{1}{2}\delta \right), & \mathrm{otherwise} \end{array}\right.$$

Here, *y* denotes the true survival in days, $\hat{y}$ the model’s predicted survival, and δ the error threshold of the Huber loss. This study sets δ=365, taking one year as the scale at which the loss switches regimes. Once training is complete, the mean absolute error (MAE) is used as the evaluation metric for survival prediction.

#### 2.8.3. Reproducibility: Random Seeds and Deterministic Settings

The patient-level split was drawn once with the seed 42 (Section 2.1.3). Both segmentation models were then trained with the global seed 1337, which was used for weight initialization, for data-loader shuffling, and for dropout; the survival models were trained without a fixed seed. The training seed was set for the Python random module, for NumPy, and for the PyTorch CPU and CUDA random number generators; torch.backends.cudnn.deterministic was set to True and torch.backends.cudnn.benchmark to False; and the data loaders were configured with a fixed worker seed. Because no seed was fixed for the survival training runs, their weight initialization and data-loader shuffling differ between runs, the survival results reported in Section 3.3.2 cannot be reproduced exactly from the released configuration, and their run-to-run variability is unknown.

#### 2.8.4. Model Selection and Hyperparameter Tuning

The 47 test patients were not used for model selection at any point; they were used once, for the final evaluation reported in Section 3.2 and Section 3.3.2. All architectural and optimization choices—the number and width of the 3D convolutional blocks, the clinical MLP widths, the dropout rate of 0.3, the learning rate and its schedule, the batch and gradient-accumulation configuration, the Huber threshold δ=365 days, the number of training epochs, and the early-stopping criterion—were fixed using the 188 training patients only. The four ablation configurations share identical values for every one of these hyperparameters and differ only in whether age and resection status are supplied, so that the ablation comparison is not confounded by tuning.

The complete hyperparameter specification of both stages is given in Table 4, the complete configuration, including the settings ordinarily left implicit, is given in Table S2 of the Supplementary Materials Section S2.

### 2.9. Model Explainability Analysis (xAI)

To improve the transparency and clinical trustworthiness of the segmentation model, this study incorporates the Gradient SHAP method to analyze the decision basis of CSFINet. Because a model that outputs only a segmentation result cannot account for the image features underlying its tumor identification, attribution analysis is used to converts the model’s judgment into a visual heatmap, enabling examination of whether the model focuses on clinically meaningful lesion regions.

Visual inspection of heatmaps is not by itself an evaluation of attribution quality. Four quantitative measures are therefore computed over all 47 test patients and reported in Section 3.4.2. (i) *Attribution mass inside the lesion*: the fraction of the total positive Gradient SHAP attribution that falls within the ground-truth whole-tumor mask, with the relative area of the mask serving as the chance-level reference. (ii) *Pointing-game accuracy*: the proportion of analyzed slices whose single highest-attribution pixel lies inside the ground-truth mask. (iii) *Deletion and insertion curves* and their areas under the curve, obtained by progressively removing or restoring the most highly attributed pixels and recording the resulting change in the model output [48]; these measure whether the highlighted pixels actually drive the prediction rather than merely coinciding with the lesion. (iv) A *sanity check* in the form of the cascading model-parameter randomization test of Adebayo et al. [38]: an attribution map that does not change as the trained weights are progressively randomized is measuring the input rather than the model.

In addition, the Spearman correlation between the attribution mass inside the lesion and the per-patient Dice coefficient is reported, which quantifies the qualitative observation that attribution becomes more diffuse in poorly segmented cases. These measures are used as plausibility checks on the model. Saliency and attribution methods have been shown to localize unreliably in medical imaging [39], and the attribution maps presented in this study are therefore not evidence of clinical validity.

## 3. Results

This section reports the experimental setup, the training environment, and the results. It introduces the hardware and software configuration, then analyzes brain tumor segmentation and survival prediction results in turn, evaluating the segmentation model by means of the Dice coefficient, precision, and recall.

For the survival prediction task, error distributions are used to assess prediction range and directional bias. Gradient SHAP analysis then examines the image locations attended to by the segmentation model, and case studies illustrate the consistency between model predictions and ground-truth annotations. Through these analyses, this section provides an overall assessment of the proposed two-stage model in terms of image segmentation, survival prediction, and model explainability.

### 3.1. Experimental Environment

All model training and testing in this study were conducted under the Windows 10 64-bit operating system. The overall configuration of the experimental environment is summarized in Table 5.

The training data were drawn from the BraTS 2020 dataset, from which 235 cases with complete survival labels were retained. The experiments use 80% of the data as the training set and 20% as the testing set: the images of 47 patients were held out as testing data and the remaining 188 patients were used as training data. To ensure methodological rigor, all slices belonging to the same patient were strictly assigned to the same set, thereby avoiding data leakage. The split procedure, the random seed, the patient-level unit of randomization, and the resulting distribution of resection status are described in detail in Section 2.1.3.

All results reported in this study were obtained from a single training run on a single 80:20 patient-level split with a single random seed. They therefore characterize this particular split and are not estimates of the expected performance across splits or across random initializations. The standard deviations and confidence intervals reported in the tables below quantify variation *between patients* within this test set; they do not quantify variation between splits, and the two should not be confused. Repeated *k*-fold cross-validation, which would quantify the latter [49], could not be completed within the revision period and is listed as the first methodological item of Future Work.

### 3.2. Segmentation Evaluation Metrics and Overall Results

This study evaluates the CSFINet segmentation model on the 47 test patients. Since the BraTS 2020 annotations comprise three tumor regions—necrotic and non-enhancing tumor core (NCR/NET), peritumoral edema (ED), and enhancing tumor (ET)—these are merged into the whole tumor (WT) region for evaluation.

For evaluation, NCR/NET, ED, and ET are treated as a unified tumor foreground in both the ground-truth annotations and model predictions, and the resulting binary whole-tumor mask is compared with the ground truth. The whole-tumor region is also the quantity that is passed to the second stage, for the reasons set out in Section 2.5. Following the standard BraTS evaluation convention [14,15], however, the revised Table 6 additionally reports Dice, precision, and recall separately for the tumor core (TC, defined as NCR/NET ∪ ET) and for the enhancing tumor (ET). These subregion metrics were computed from the stored four-class predictions of the already-trained models and required no retraining.

To quantify the similarity between the predicted tumor mask and the ground-truth mask, the Dice coefficient, precision, and recall are adopted as the principal evaluation metrics. All three are computed from the true positives (TP), false positives (FP), and false negatives (FN) of the confusion matrix.

The Dice coefficient measures overlap between the predicted region and the true tumor region; a higher value indicates closer agreement with the manual annotation. Precision measures the proportion of the predicted tumor region that truly belongs to the tumor, while recall measures the proportion of the true tumor region successfully detected by the model.

The metrics are computed as follows:

(23) $$Dice=\frac{2TP}{2TP+FP+FN}$$

(24) $$Precision=\frac{TP}{TP+FP}$$

(25) $$Recall=\frac{TP}{TP+FN}$$

Here, TP denotes the number of pixels correctly predicted as tumor; FP the number of pixels incorrectly predicted as tumor; and FN the number of pixels that are truly tumor but were not detected by the model.

To verify the improvement of CSFINet over conventional convolutional segmentation models, U-Net was additionally used as a baseline for comparison. Both models use the same test data, image preprocessing, and training settings, so as to ensure a fair comparison. The baseline is deliberately constrained to exactly the training configuration of CSFINet—2D slice-wise training on a single 12 GB GPU, plain multi-class cross-entropy without a Dice term, no data augmentation, no ensembling, no test-time augmentation, and no post-processing—so that the comparison isolates the effect of the architectural change. Both models were trained for the same fixed budget of 24 epochs, and the early-stopping criterion was not triggered for either. It is referred to below as “U-Net (baseline, identical training configuration)” and is *not* representative of the performance of a fully configured U-Net; published 3D U-Net implementations reach a whole-tumor Dice of approximately 0.84 on BraTS 2020 [25]. Section 3.2 places the present results in the context of the published literature.

All metrics are reported as the mean ± standard deviation of the per-patient value over the 47 test patients, together with the median and interquartile range (which is more informative than the mean for the strongly left-skewed Dice distribution) and a 95% confidence interval of the mean obtained from 10,000 bias-corrected and accelerated (BCa) bootstrap resamples of the patient-level values [50]. The paired difference in per-patient Dice between CSFINet and the U-Net baseline is reported with a 95% bootstrap confidence interval and a Wilcoxon signed-rank *p* value [51].

The average segmentation results over the 47 test patients are presented in Table 6.

As Table 6 shows, CSFINet improves substantially on the U-Net baseline in Dice and in recall, whereas the two models are comparable in precision. The mean Dice increases from 0.5084 for U-Net to 0.7849 for CSFINet, a paired difference of +0.2766 (95% CI 0.2304–0.3334, $p=3.5\times 10^{-9}$), and the mean recall increases from 0.3822 to 0.7480, a paired difference of +0.3658 (95% CI 0.3257–0.4140). The mean precision increases only from 0.8676 to 0.8739; the paired difference of +0.0063 has a 95% confidence interval of −0.0601 to 0.1086, which includes zero, so no difference in precision is established by these data. The claim of improvement therefore rests on Dice and recall, not on precision.

CSFINet improves brain tumor segmentation in overlap and in sensitivity, while leaving precision essentially unchanged. The improvement in mean Dice indicates greater overlap between the predicted and ground-truth regions, suggesting that the cross-scale feature interaction and alignment mechanisms enhance the model’s ability to learn tumor features across multiple scales.

CSFINet also attains a high mean precision (0.8739); this value is comparable to that of the baseline (0.8676) rather than an improvement over it, and it indicates that the predicted tumor regions contain few false positives in both models. What distinguishes CSFINet is that it reaches this precision while detecting far more of the tumor: its mean recall of 0.7480, against 0.3822 for the baseline, is the source of the gain in Dice. Recall remains lower than precision, indicating that some small or indistinctly bounded tumor regions still go undetected.

In summary, CSFINet achieves a mean whole-tumor Dice of 0.7849, an improvement of 0.2766 over a U-Net trained under an identical training configuration. This comparison establishes the effect of the cross-scale feature interaction and alignment mechanisms under a fixed, constrained training configuration; it does not establish that CSFINet is competitive with the current state of the art, which is addressed in Section 3.2. Every improvement figure quoted in this section is relative to that in-house baseline, and none of them is relative to the published results collected in Table 2. These segmentation results provide the whole-tumor masks that are used as the image input of the subsequent survival prediction model.

#### Comparison with the Current Literature

A comparison against a single 2015 U-Net baseline is not sufficient to situate the present results within the field. Retraining nnU-Net, UNETR, or Swin UNETR under the hardware constraints of this study (a single 12 GB GPU, 2D slice-wise training) would not produce a fair representation of those methods either. Table 2 therefore reports published BraTS 2020 results for a set of representative architectures alongside the present results, and the values for the other methods are taken directly from the cited publications.

Four reasons prevent these figures from being read as a controlled comparison. First, the cited works evaluate on the official BraTS validation or test partitions through the challenge server, whereas the present study evaluates on its own 47-patient hold-out drawn from the training release. Second, they train and evaluate on full three-dimensional volumes, whereas the hardware available here forced 2D slice-wise training and slice-wise evaluation. Third, most of them use ensembling, test-time augmentation, extensive data augmentation, and compound Dice + cross-entropy losses, whereas the present models use a single run, no augmentation, and cross-entropy only. Fourth, the U-Net baseline reported here was deliberately constrained to the training budget of CSFINet, which is precisely why it does not represent a properly tuned U-Net. Table 2 is therefore provided for context only and the segmentation improvement reported in this article is defined solely against the U-Net baseline trained here. The segmentation performance reported in this study is accordingly below the current state of the art on BraTS 2020, and closing that gap—through fully three-dimensional training, a compound loss, data augmentation, and a comparison against a properly configured nnU-Net—is listed as the first item of Future Work.

### 3.3. Survival Prediction Results and Analysis of Clinical Factors

To investigate the influence of age and resection status on survival prediction, this study compares four input feature configurations: image only, image combined with age, image combined with resection status, and image combined with both age and resection status. The evaluation metrics for survival prediction are introduced first, followed by a comparison of the four ablation results.

#### 3.3.1. Evaluation Metrics for Survival Prediction

This study evaluates survival prediction performance using the mean absolute error (MAE), root mean squared error (RMSE), median absolute error (median AE), the Pearson correlation coefficient, and the Spearman rank correlation coefficient.

The MAE denotes the mean absolute difference between the predicted and true survival; a lower value indicates a smaller average prediction error. The RMSE penalizes larger prediction errors more heavily and can therefore indicate whether the model exhibits pronounced outlying errors. The median AE denotes the median of all absolute errors and is less susceptible than the mean to the influence of a small number of extreme cases.

The Pearson correlation coefficient assesses the degree of linear correlation between predicted and true survival, while the Spearman rank correlation coefficient measures the monotonic relationship between their rankings. The closer either coefficient is to 1, the better the model reflects the variation in patient survival.

#### 3.3.2. Ablation Results for the Different Input Feature Configurations

To evaluate the auxiliary value of age and resection status for survival prediction, this study compares four input feature configurations under identical data splits and image processing; the results are presented in Table 7. Two reference models have been added so that the contribution of the model itself can be judged: a constant predictor that always returns the mean survival of the 188 training patients, and an ordinary least-squares regression of survival on age alone, fitted on the training patients and evaluated on the same 47 test patients. All error metrics are reported with 95% bootstrap confidence intervals (10,000 resamples) [50], and the correlation coefficients with 95% confidence intervals. As set out in Section 2.5, all four configurations were trained on ground-truth whole-tumor masks and are evaluated here on CSFINet-predicted masks, so the errors in Table 7 include the segmentation error of the first stage. The survival training runs were not seeded (Section 2.8.3), so these values are those of one unseeded run and cannot be reproduced exactly.

As Table 7 shows, the model combining imaging with age achieves the lowest MAE and RMSE among the four configurations, with an MAE, RMSE, and median AE of 251.69 days, 361.78 days, and 175.69 days, respectively, all lower than those of the image-only model; the lowest median absolute error, 171.22 days, is that of the model combining imaging with resection status. Its Pearson and Spearman rank correlation coefficients are likewise the highest among the four models, at 0.471 and 0.462, respectively.

These results reflect a comparison under the present dataset and validation sample only and are insufficient to establish statistically significant differences among the models.

#### 3.3.3. Resection Status and the Distribution of Absolute Error

**Figure 8:**
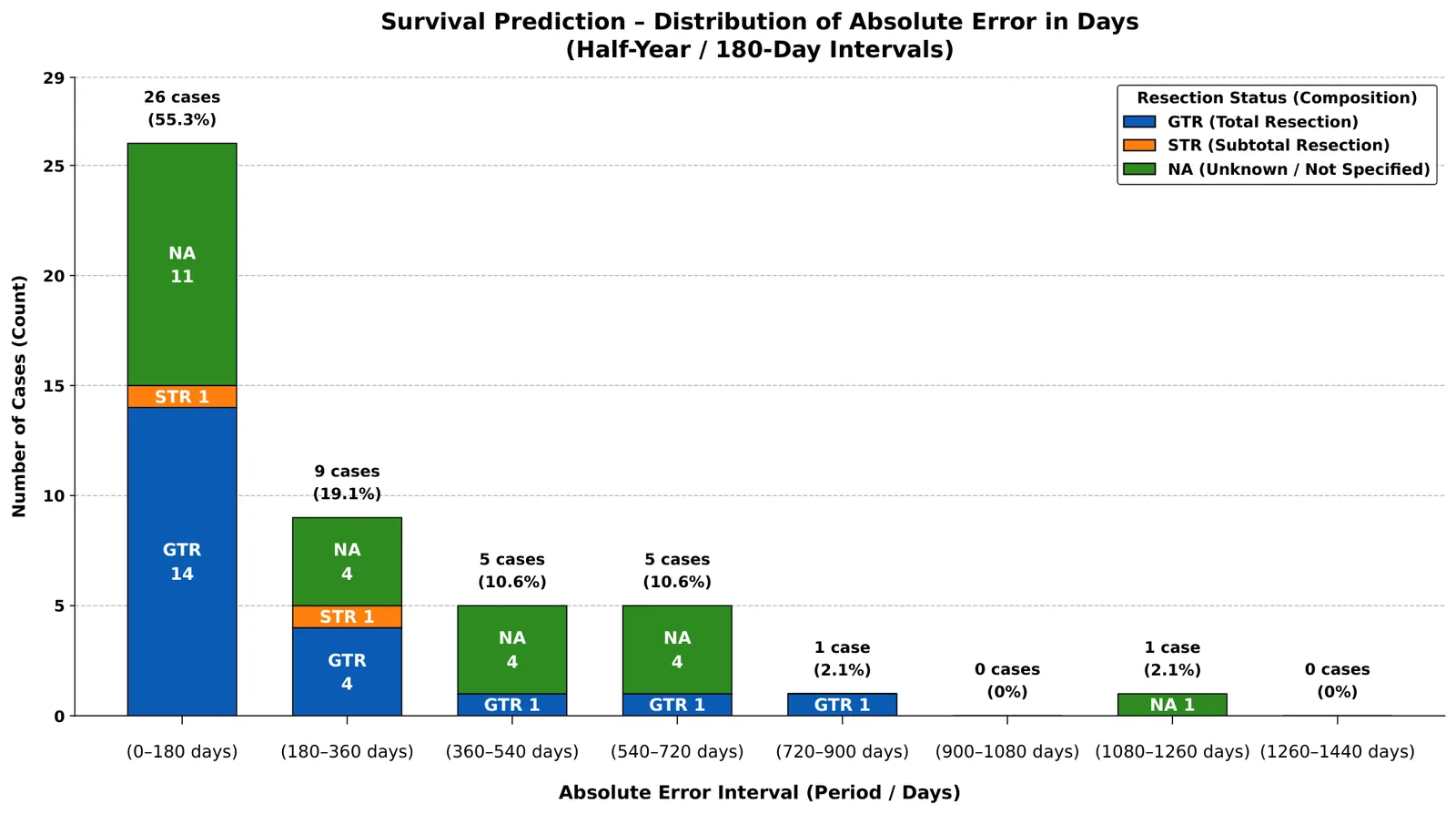
Distribution of absolute error in days for the model combining imaging with age, over the 47 test patients, stratified by resection status (GTR n=21, STR n=2, not recorded n=24). Errors are grouped in explicit 180-day intervals (0–180, 181–360, 361–540, 541–720, and >720 days), and the number of patients in each interval is given above the corresponding bar. Resection status is used here only to describe the composition of the error groups and is not an input feature of this model.

Since the model combining imaging with age achieved the best overall performance, it was chosen as the principal model for the subsequent analyses of prediction error, residual distribution, and associations with clinical factors. To examine how error distribution varies with resection status, patients were stratified—based on the resection status recorded in the validation data—into three groups: gross total resection (GTR), subtotal resection (STR), and incomplete data (N/A), and absolute errors were tabulated at 180-day intervals, as shown in Figure 8.

It should be noted that resection status is not an input feature of the model combining imaging with age; it is used here only for a grouped error analysis after prediction, and does not imply that the model used resection status information during prediction.

As Figure 8 shows, among the 47 validation patients, 26 (55.3%) have an absolute error between 0 and 180 days, and a further 9 (19.1%) have an absolute error between 180 and 360 days; in total, 35 patients (74.5%) have a prediction error within 360 days.

In addition, 5 patients (10.6%) have an absolute error between 360 and 540 days, and the remaining cases have errors exceeding 540 days, indicating that the model still struggles with patients with more extreme survival. Since the STR group contains markedly fewer samples than the GTR and N/A groups, this figure serves principally to present the error composition across resection statuses and should not be taken as evidence of significant performance differences among the resection status groups.

#### 3.3.4. Prediction Residuals and the Distribution of Over- and Underestimation

**Figure 9:**
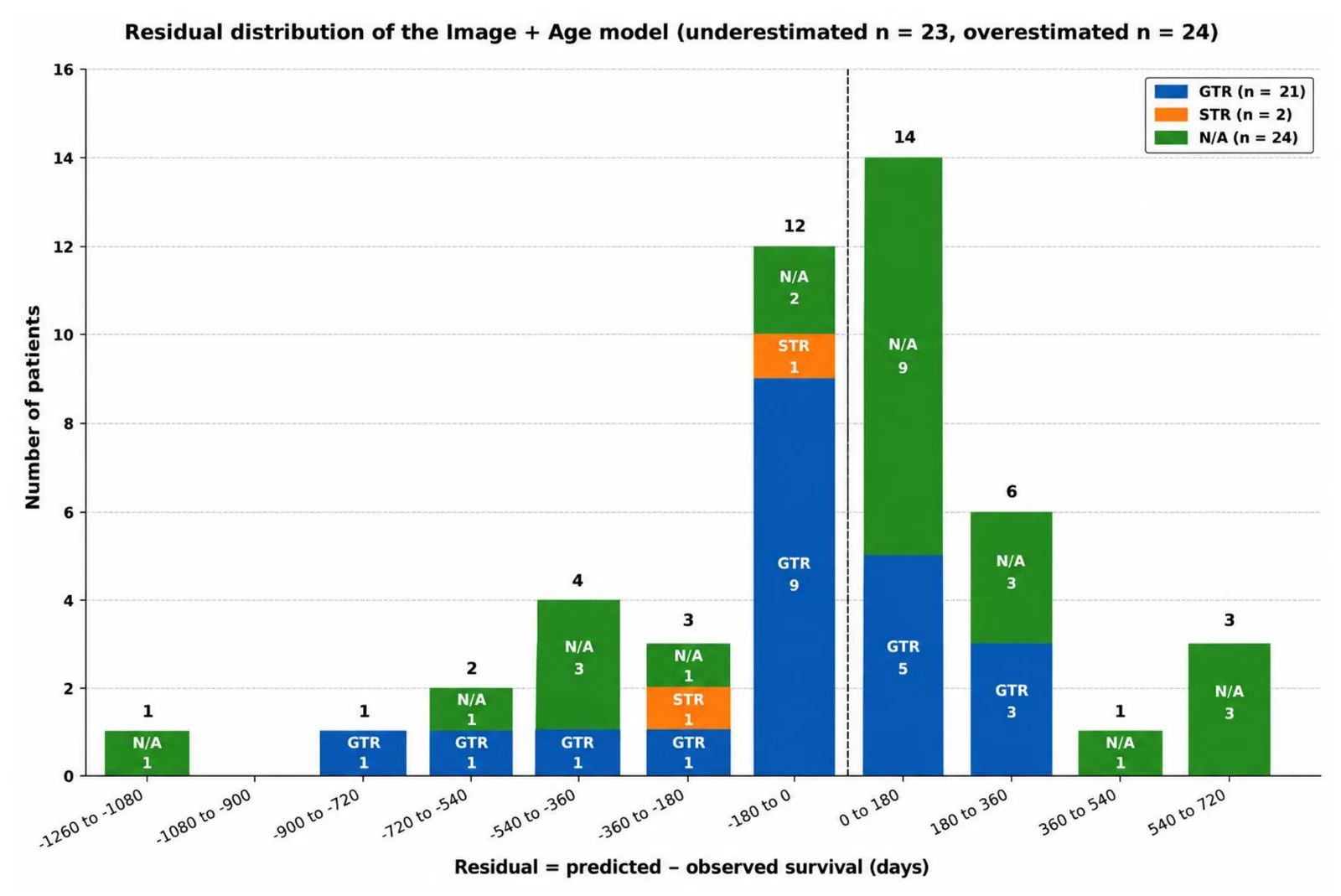
Prediction residuals and the distribution of over- and underestimation for the model combining imaging with age, over the 47 test patients. Residual intervals are labeled by their explicit day ranges, and the number of patients in each interval is given above the corresponding bar.

To analyze the model’s tendency toward overestimation and underestimation further, the prediction residual is defined as:

(26) $$e_{i}={\hat{y}}_{i}-y_{i}$$

where ${\hat{y}}_{i}$ denotes the predicted survival of the *i*-th patient and $y_{i}$ the true survival. A residual greater than 0 indicates that the model overestimates the patient’s survival, while a residual less than 0 indicates underestimation. Residuals are likewise tabulated in 180-day intervals; the results are shown in Figure 9.

As Figure 9 shows, among the 47 validation patients, the model combining imaging with age underestimates survival for 23 patients and overestimates it for 24, indicating that the model is not markedly biased in a single direction.

However, the magnitude of the residuals in underestimated cases is considerably larger than in the overestimated cases, with the greatest underestimation reaching 1080 to 1260 days. This suggests that the model may tend to predict values closer to the dataset mean for patients with longer true survival, reflecting a possible regression toward the mean. Resection status is used here only to present patient composition within each residual interval and is not an input feature of the model.

#### 3.3.5. Relationship Between Age and True Survival

**Figure 10:**
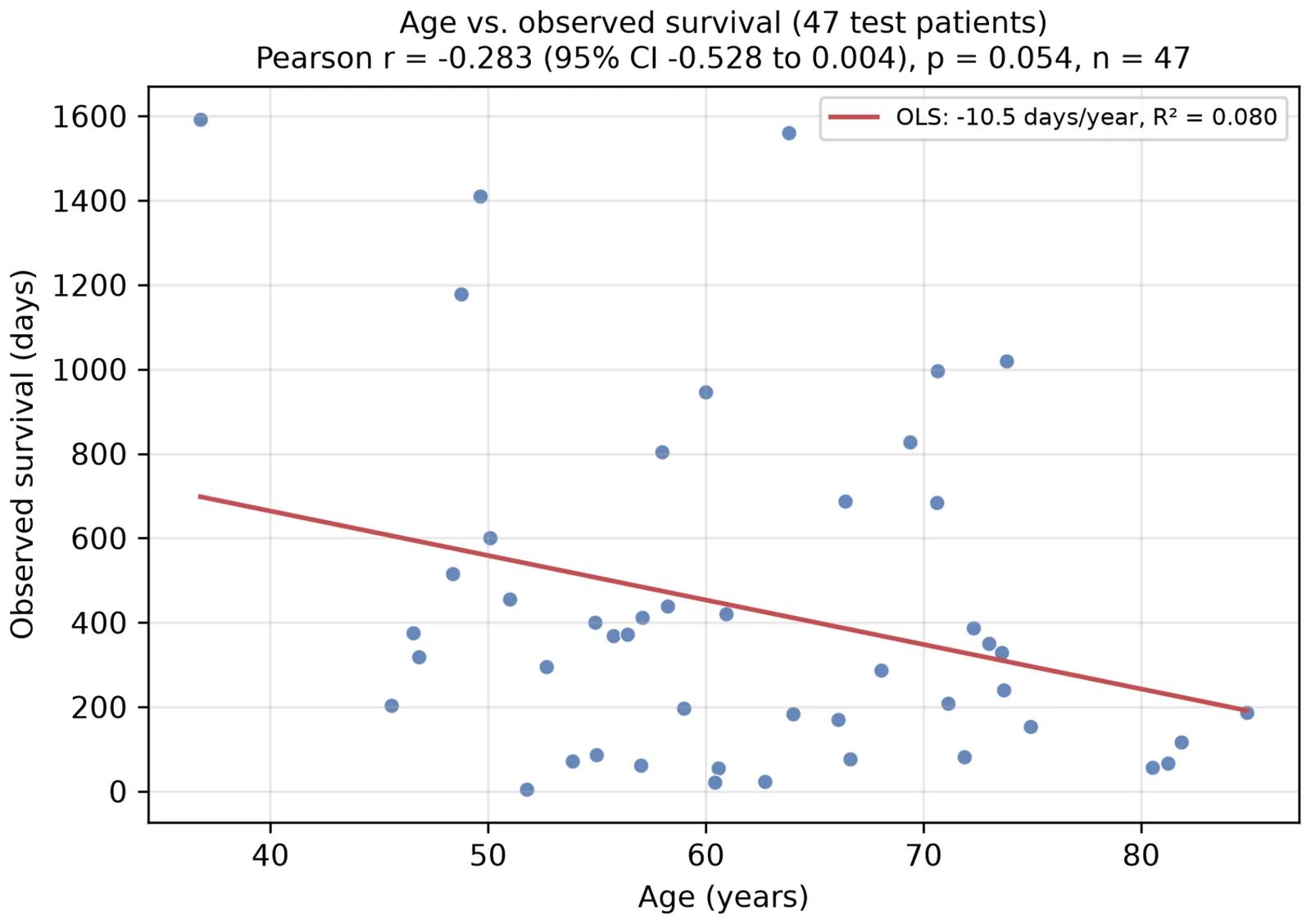
Relationship between age and true survival, among the 47 test patients (Pearson r=−0.283, 95% CI −0.528 to 0.004, p=0.054). Exploratory analysis of a single test set; the fitted line describes association only and does not imply a causal effect of age on survival.

To analyze the relationship between the age of the validation patients and their true survival, a scatter plot of age against true survival was produced, together with the Pearson correlation coefficient and a linear regression trend; the results are shown in Figure 10. This analysis and that of Section 3.3.6 are restricted to the 47 test patients, are exploratory in nature, are not adjusted for multiple comparisons, and describe association only; they do not indicate a causal relationship between age and survival.

As Figure 10 shows, the Pearson correlation coefficient between age and true survival is r=−0.283 (95% CI −0.528 to 0.004), indicating a weak negative correlation; the slope of the linear regression corresponds to an average decrease of 10.5 days in true survival per additional year of age. However, the *p* value is 0.054, slightly above the conventional significance level of 0.05, so that the linear relationship between age and true survival cannot be regarded as statistically significant in this validation set.

Moreover, $R^{2}=0.080$ for this linear model, indicating that age accounts for only part of the variation in survival—patient survival is likely jointly influenced by tumor characteristics, treatment regimen, molecular markers, and general health status.

#### 3.3.6. Relationship Between Age and Predicted Survival

**Figure 11:**
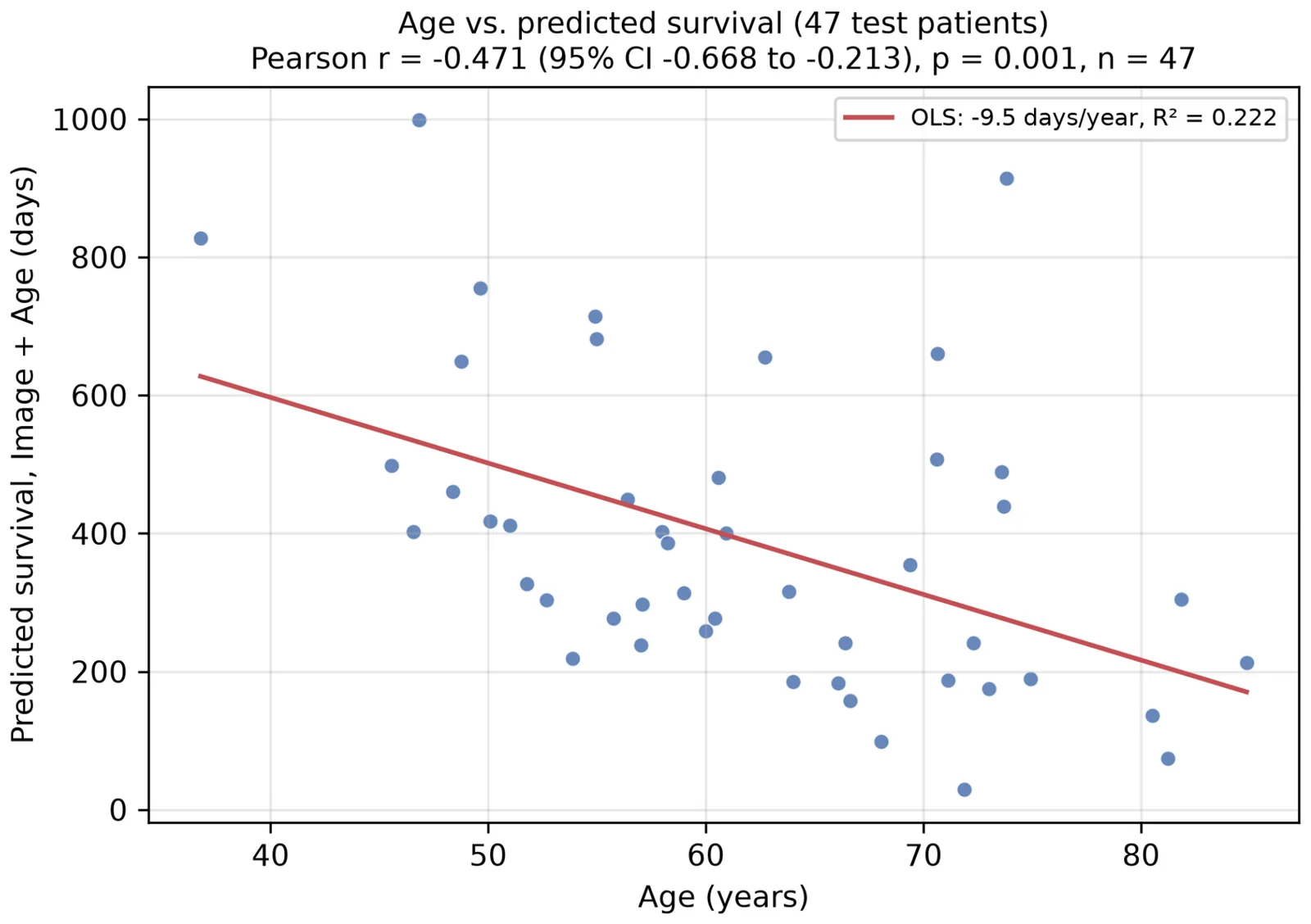
Relationship between age and the survival predicted by the model combining imaging with age, among the 47 test patients (Pearson r=−0.471, 95% CI −0.668 to −0.213, p=0.001). Exploratory analysis; a stronger association with predicted than with observed survival may indicate over-reliance of the model on age (see Section 4.1) and does not indicate a causal relationship.

To examine the relationship between age and the predictions produced by the model combining imaging with age, a scatter plot of age against predicted survival was additionally produced; the results are shown in Figure 11.

As Figure 11 shows, the Pearson correlation coefficient between age and the model’s predicted survival is r=−0.471 (95% CI −0.668 to −0.213) with p=0.001, indicating a statistically significant moderate negative correlation. The slope of the linear regression corresponds to a decrease of 9.5 days in predicted survival per additional year of age, with $R^{2}=0.222$.

By comparison, the correlation between age and true survival is only r=−0.283, indicating that age correlates more strongly with predicted than with true survival. This pattern is examined as a potential indication of over-reliance on age, rather than as evidence of the usefulness of age, in Section 4.1. The addition of age was associated with a lower overall prediction error in this cohort, but this result must not be interpreted as a causal relationship between age and survival.

### 3.4. Case Analysis

To examine model performance at the level of individual patients, three representative cases were selected. One representative case (BraTS20_199, a high-Dice case) is presented in full below; the remaining two cases—BraTS20_070, a case of lower segmentation quality, and BraTS20_094, a case of moderate segmentation quality—are presented in Section S5 of the Supplementary Materials. Each case presents the slice-level distribution of tumor pixels, segmentation results for representative MRI slices, and the survival predicted by the model combining imaging with age. Comparing ground-truth annotations with CSFINet predictions examines segmentation capability across different tumor locations, sizes, and morphologies, while comparing true and predicted survival reveals the relationship between segmentation quality and downstream survival prediction.

In the slice-level analysis, the number of true tumor pixels per slice is compared with the model’s predictions, together with the variation in the Dice coefficient across slices. Survival results are taken from the model combining imaging with age, which achieved the best overall performance in the ablation study. (Resection status is presented as background information only and is not an input feature of that model.)

#### 3.4.1. Case 2: BraTS20_199

The patient in this case is 69.364 years of age, with an unknown or unlabeled resection status (N/A). The true survival is 828 days, and the survival predicted by the model combining imaging with age is 354.481 days, an underestimate of 473.519 days relative to the true value. The Dice coefficient of the segmentation result is approximately 0.909, making this a case of good segmentation performance. The basic information for this case is presented in Table 8.

**Figure 12:**
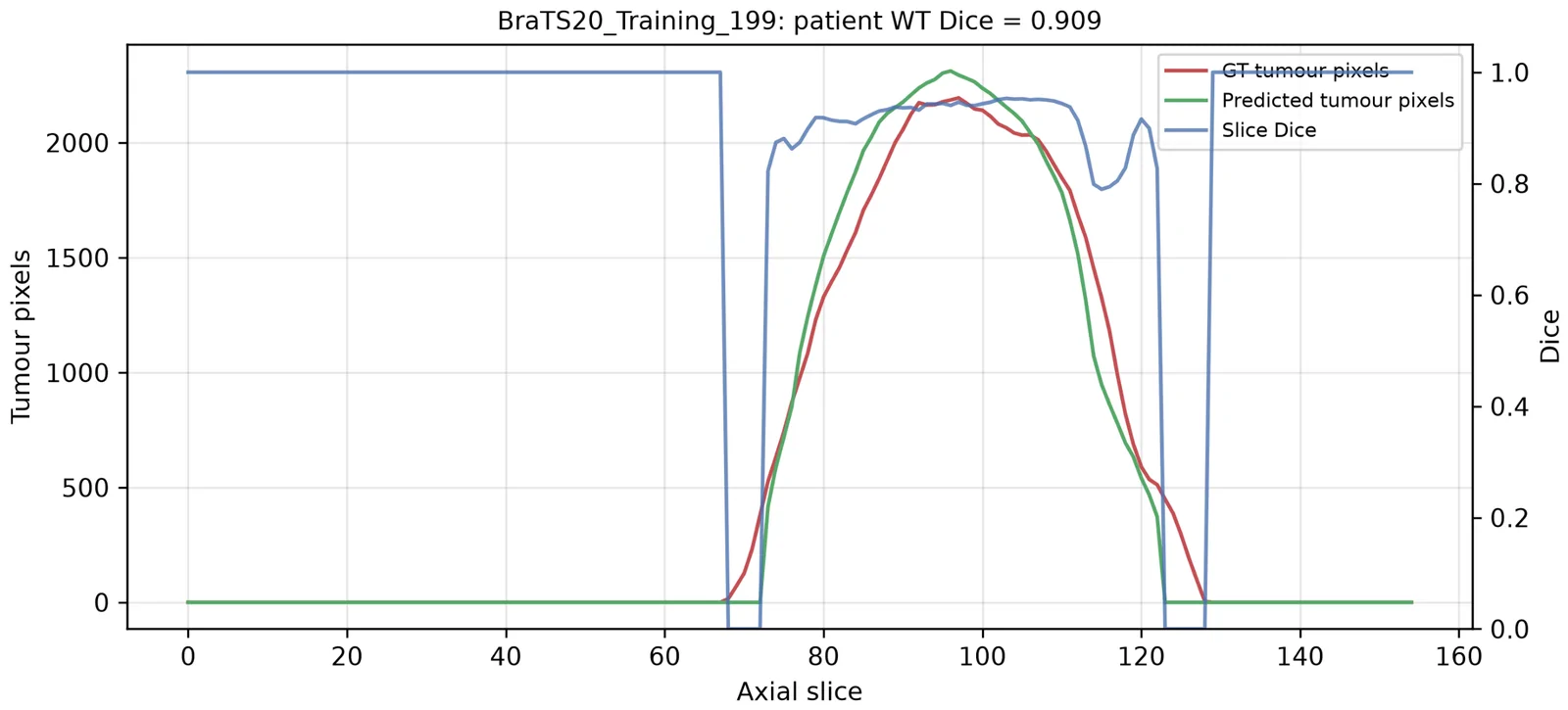
Distribution of tumor pixels and variation in the Dice coefficient for BraTS20_199.

Figure 12 shows the slice-level distribution of tumor pixels and the variation in the Dice coefficient for BraTS20_199. The number of tumor pixels predicted by the model and the number in the ground truth follow highly similar trends over the principal tumor range, and the two curves largely coincide. This indicates that the model accurately captures the slice range in which the tumor appears and effectively predicts the variation in tumor area across slices.

The patient Dice for this case reaches 0.909, indicating a high degree of overlap between the predicted mask and the ground-truth annotation. Compared with Case 1 (c.f., Section S5 of the Supplementary Materials), the predictions in this case are closer to the ground truth in both tumor extent and slice distribution, showing that CSFINet segments well those cases in which the tumor boundary is more distinct and the tumor more spatially concentrated.

**Figure 13:**
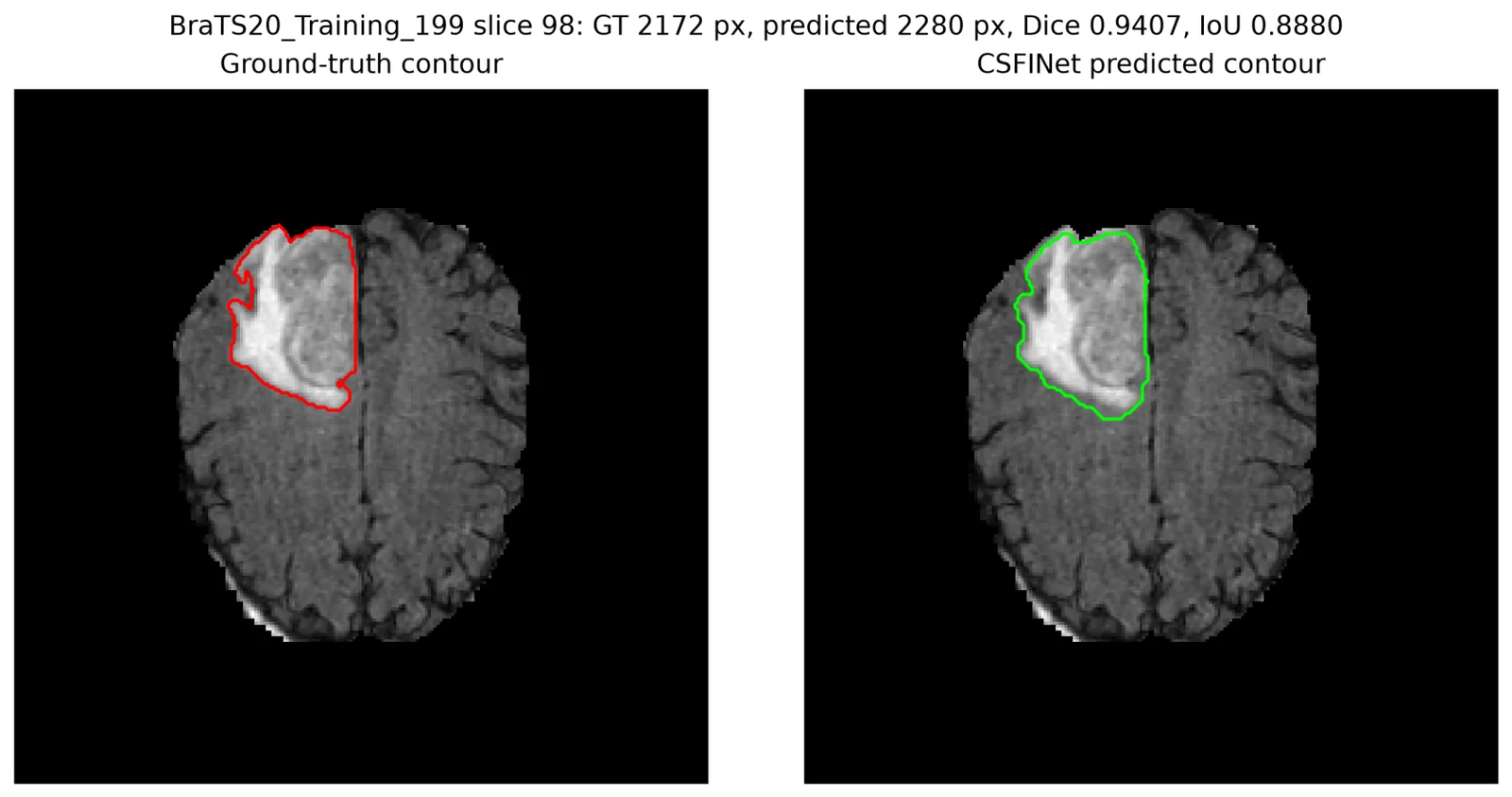
Comparison of segmentation results for slice 98 of BraTS20_199. (**Left**) Ground-truth whole-tumor contour, shown in red; (**right**) whole-tumor contour predicted by CSFINet, shown in green.

Figure 13 shows the segmentation result for slice 98 of BraTS20_199. The left panel shows the ground-truth tumor contour and the right panel the model’s predicted contour. The predicted contour overlaps the ground-truth annotation to a high degree and covers the principal tumor region completely, indicating good tumor localization and boundary prediction on this slice.

**Figure 14:**
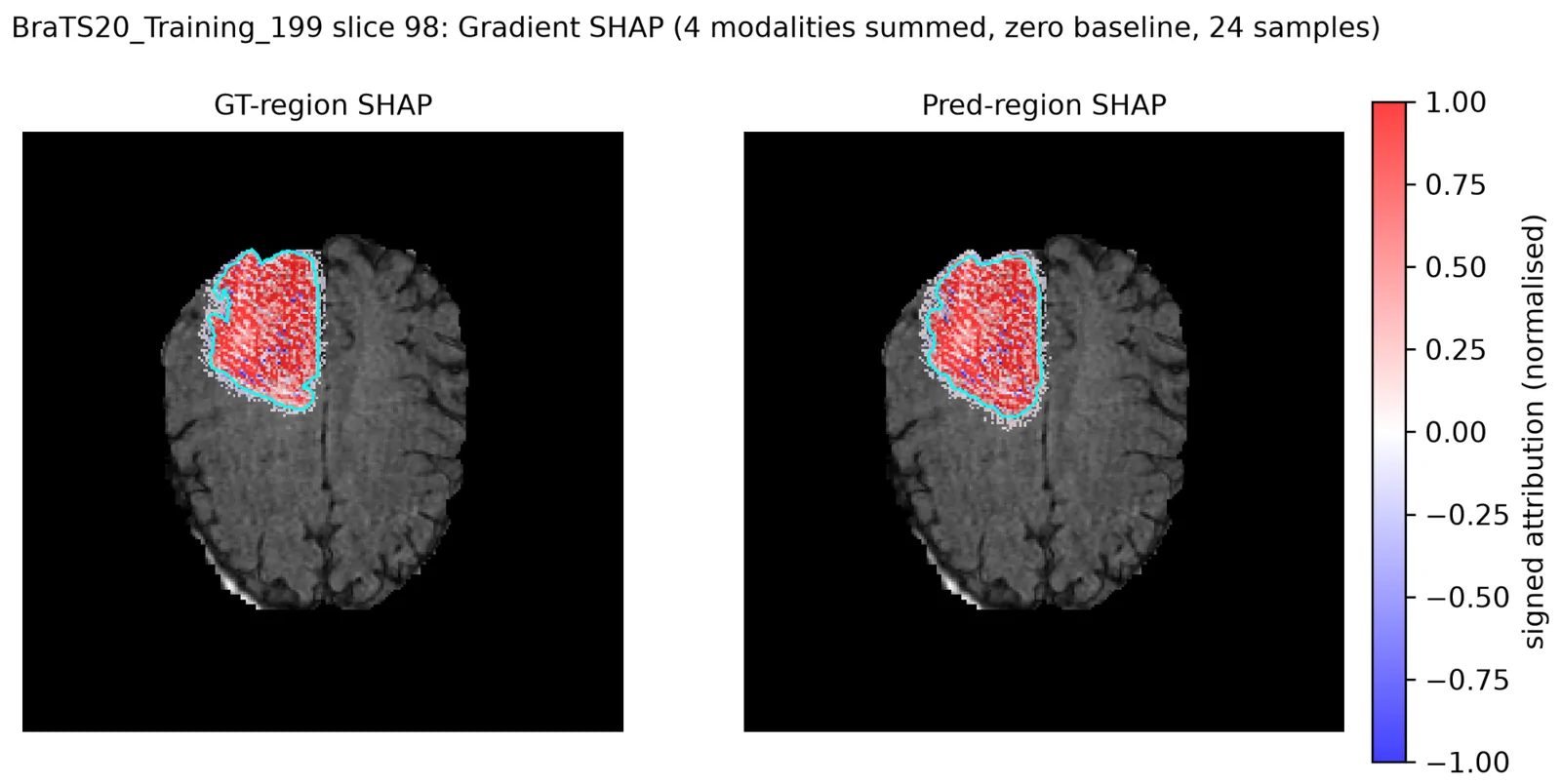
Gradient SHAP explainability analysis for slice 98 of BraTS20_199, with attributions summed over the four modalities (zero baseline, 24 samples). (**Left**) Attribution map for the ground-truth tumor region; (**right**) attribution map for the CSFINet-predicted tumor region. The cyan line outlines the ground-truth whole-tumor contour in the left panel and the predicted whole-tumor contour in the right panel. Red denotes positive and blue negative attribution toward the tumor prediction, normalized to the range −1 to 1.

To examine further the image regions on which the model based its segmentation of this slice, a Gradient SHAP explainability analysis was performed on slice 98 of BraTS20_199. Figure 14 shows the SHAP attribution results for the GT region and the predicted region, in which red denotes image locations contributing positively to the model’s tumor prediction and blue denotes locations with a negative influence on that prediction.

The figure shows that, whether the ground-truth region or the model’s predicted region is used as the analysis target, the regions of high attribution are concentrated principally at the tumor location and overlap the tumor contour to a high degree. This indicates that, in this case, the model bases its tumor segmentation chiefly on the image features of the lesion itself rather than being influenced by the background or by unrelated tissue. This is consistent with the relatively high Dice coefficient of this case, showing that the model’s segmentation not only agrees closely with the ground-truth annotation but also rests on a reasonably plausible decision basis.

This case shows that CSFINet accurately predicts the location and extent of the tumor, and the Gradient SHAP results indicate that the model attends principally to the tumor lesion. Nevertheless, despite a Dice coefficient of ~0.909, the model still underestimates survival by 473.519 days. This indicates that high segmentation quality does not necessarily guarantee accurate survival prediction, since prognosis may also be influenced by factors not included in the model, such as treatment response, molecular markers, and general health status.

#### 3.4.2. Quantitative Assessment of Attribution Quality

The case studies above illustrate the attribution maps visually, but visual similarity between a heatmap and a lesion is not an evaluation. The four measures defined in Section 2.9 were therefore computed over all 47 test patients, rather than on the three illustrative cases alone; the results are summarized in Table 9. The attribution mass inside the lesion is compared against the relative area of the ground-truth mask, which is the value expected from a spatially uninformative attribution map; the deletion and insertion areas under the curve indicate whether the highlighted pixels actually drive the model output [48]; and the model-parameter randomization test verifies that the attribution maps degrade as the trained weights are randomized, which is the minimum requirement for an attribution method to be measuring the model at all [38].

The Spearman correlation between the attribution mass inside the lesion and the per-patient Dice coefficient is ρ=0.405 (p=0.005), which quantifies the qualitative observation reported in the case studies that attribution becomes more diffuse when segmentation quality is lower. These measures characterize the internal consistency of the model and its explanations; they are not evidence that the attribution maps identify clinically meaningful structures, since saliency methods have been shown to localize unreliably in medical imaging [39].

### 3.5. Web-Based Brain Tumor Analysis and Visualization System

An interactive web application integrating the brain tumor segmentation, survival prediction, and model explainability results was developed using Python and Streamlit Version 1.52.0, available at https://csfi-bmmissp.know-eng.net/ (accessed on 28 September 2026). The application is a research prototype intended for the visual inspection and error analysis of model outputs. It has not been evaluated with clinical users, has not undergone any usability or safety assessment, has not been validated within a clinical workflow, and is not a medical device; it holds no regulatory clearance in any jurisdiction. Its outputs, including the predicted survival values and the attribution maps, must not be used to inform patient management. The application brings together patient clinical data, survival predictions, the tumor slice range, segmentation evaluation metrics, and Gradient SHAP attribution results within a single interface, enabling users to inspect model outputs at both the patient and slice levels.

**Figure 15:**
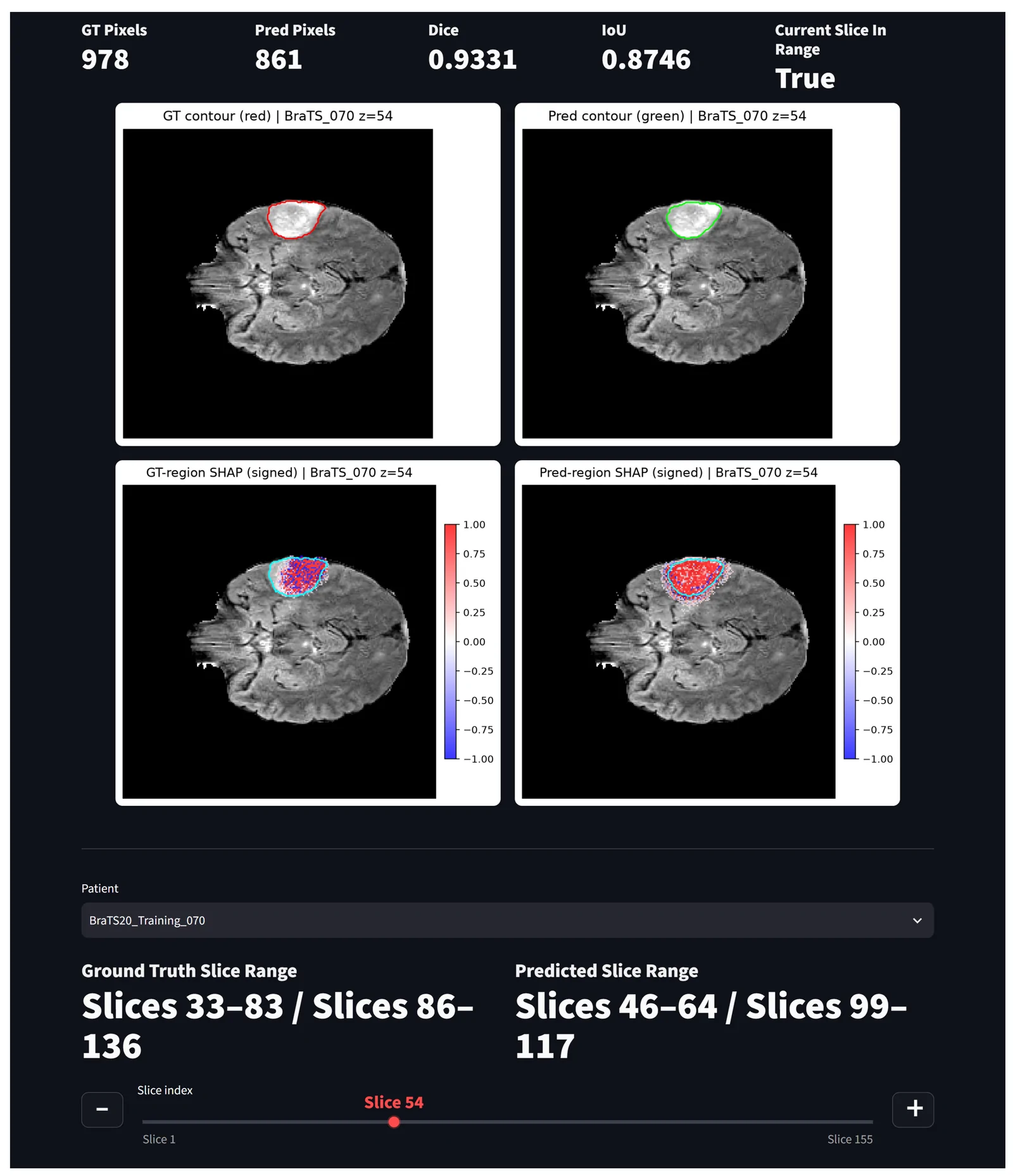
Tumor segmentation contours and Gradient SHAP attribution results for slice 54 of BraTS20_Training_070.

The complete step-by-step walkthrough of the interface—patient selection, the prediction and slice-control panels, the modality and ablation menus, and the slice-level comparison of segmentation contours with Gradient SHAP attribution—is provided in Section S5 of the Supplementary Materials. Figure 15 shows a representative view of the interface: the segmentation contours and the attribution map for a single slice.

In summary, the web application developed in this study presents survival and overall tumor segmentation results at the patient level, and additionally allows the segmentation contours, evaluation metrics, and model attribution distribution of individual slices to be inspected via the slice slider. The system serves principally as a tool for presenting research results, comparing cases, and analyzing model errors. It has not been validated in a clinical setting, and its outputs are therefore intended for research analysis only and should not be used directly as a basis for clinical diagnosis or treatment decisions.

## 4. Discussion

This section examines whether the survival model is driven by the imaging input or by age, and then states the limitations of the study. Both discussions are framed by the fact that all results originate from a single split of a single retrospective cohort.

### 4.1. Is the Survival Model Imaging-Driven or Age-Driven?

Section 3.3.5 and Section 3.3.6 report that age correlates more strongly with the model’s predictions (r=−0.471) than with the survival actually observed in the same 47 patients (r=−0.283). The model therefore reproduces the age–survival relationship more strongly than that relationship exists in the observed data. This is the pattern expected when a low-dimensional, well-scaled clinical feature dominates a high-dimensional imaging feature that the model has been unable to exploit, and, taken together with the compression of the predictions toward the cohort mean documented in Section 3.3.4, it is consistent with the survival model behaving closer to an age-driven regression than to an imaging-driven one. It should therefore be read primarily as a warning sign of over-reliance on age—and possibly of overfitting—rather than as evidence of the usefulness of age.

The difference between the two correlations was tested formally. Because the two coefficients are dependent and overlapping (both involve age, and both are computed on the same patients), Steiger’s *Z* test for elements of a correlation matrix was applied [52], with r(age,observed)=−0.283, r(age,predicted)=−0.471, r(observed,predicted)=0.471, and n=47. The difference is not statistically significant (Z=1.34, p=0.18), and the two confidence intervals overlap substantially. The data are therefore equally compatible with genuine over-reliance on age and with sampling noise at this sample size, and neither interpretation can be excluded.

### 4.2. Limitations

*Study design and external validity.* This is a retrospective analysis of a single, publicly curated, multi-institutional but centrally preprocessed cohort. The 47-patient hold-out provides internal validation only; no temporal, geographical, or institutional external validation was performed. The errors reported here are therefore optimistic estimates of the error that would be observed on new data, and the ordering of the four feature configurations is specific to this dataset and this validation split. External validation on cohorts such as later BraTS releases [31], the UPenn-GBM cohort [53], and the UCSF-PDGM cohort [54] is the primary item of Future Work.

*Single split and single seed.* All results come from one training run on one 80:20 patient-level split (Section 2.8.3). The patient-level split and the two segmentation runs were seeded (42 and 1337, respectively), but the survival training runs were not seeded at all, so the survival results in Table 7 are those of a single unseeded run and cannot be reproduced exactly; their run-to-run variability is unknown and may be of the same order as the differences between the four feature configurations. The dispersion measures reported in Table 6 and Table 7 quantify between-patient variation, not between-split or between-seed variation. Repeated *k*-fold cross-validation would be required to establish the stability of these results [49] and could not be completed within the revision period.

*Statistical power.* With 47 test patients, the association analyses have 80% power only for |r|≥0.40. The analyses of Section 3.3.5 and Section 3.3.6 are exploratory, are unadjusted for multiple comparisons, and describe association rather than causation.

*Clinical interpretability of the error magnitude.* Median overall survival in the pivotal trial of radiotherapy plus concomitant and adjuvant temozolomide for glioblastoma was 14.6 months, that is, approximately 445 days [55]. A mean absolute error of 251.69 days therefore corresponds to approximately 57% of the median survival of the disease being predicted. An error of this magnitude cannot support individual prognostic counseling, treatment stratification, or any clinical decision; the value of the present results lies in the comparison between feature configurations, not in the absolute accuracy of the predictions.

*Resection status and missing data.* Encoding the unknown/unlabeled category as a third level of the one-hot vector is the missing-indicator method, and it renders the corresponding model parameter uninterpretable, because it mixes patients whose resection status was genuinely unknown with patients whose record was simply not transferred to the dataset [44,45]. The NA group is large enough that the network could exploit missingness itself as a predictive signal, which would not transfer to a cohort in which missingness arises for different reasons. The null ablation result for resection status must therefore not be read as evidence that the extent of resection is unimportant—an interpretation that would contradict the surgical literature [6,7,8,9]—but only as evidence that this three-level, partly missing label carried no usable signal in this cohort. The official BraTS overall-survival task is itself restricted to patients who underwent gross total resection [15], which further indicates that this field was not intended to serve as a modeled predictor.

*Censoring.* As described in Section 2.1.2, the released survival times carry no event indicator, so all observations are treated as complete. If some are in fact censored, the model will systematically underestimate survival for long survivors.

*Segmentation performance and baseline.* The reported segmentation performance is below the current state of the art on BraTS 2020 (Section 3.2), and the U-Net baseline is constrained to an identical training configuration and does not represent a properly configured U-Net. The comparison therefore establishes the effect of the architectural change under that configuration, not competitiveness with the state of the art.

*Binary whole-tumor input.* The survival model consumes a binary whole-tumor mask, which discards the enhancing-tumor and necrotic-core volumes that carry additional prognostic information (Section 2.5).

*Explainability.* Gradient SHAP attribution is used as a plausibility check on the segmentation model. The quantitative measures of Section 3.4.2 characterize the internal consistency of model and explanation only; they do not establish clinical validity [37,39].

*Reporting.* The study report follows the TRIPOD+AI statement for the prediction-model component [56,57] and the STROBE statement for the cohort-description component [58]; the completed checklists are provided as Tables S3–S7 of the Supplementary Materials.

## 5. Conclusions

This study established a two-stage brain tumor analysis pipeline using the BraTS 2020 multimodal MRI dataset. In the first stage, CSFINet segments the tumor regions; in the second stage, the binary whole-tumor mask serves as the image input, while patient age and resection status are adopted as candidate clinical features, giving rise to four survival prediction models: image only, image combined with age, image combined with resection status, and image combined with both age and resection status. Ablation experiments evaluate the auxiliary value of the different clinical features for survival prediction.

Regarding segmentation, U-Net was used as the baseline under identical data splits, training settings, and postprocessing. U-Net achieved mean Dice, precision, and recall of 0.5084, 0.8676, and 0.3822, respectively, whereas CSFINet attained 0.7849, 0.8739, and 0.7480—absolute differences of +0.2766, +0.0063, and +0.3658, respectively. The gains in Dice and recall are large and their paired confidence intervals exclude zero; the precision difference is small and its confidence interval (−0.0601 to 0.1086) includes zero, so the two models should be regarded as comparable in precision. The improvement in recall is the most pronounced, indicating that CSFINet detects a greater proportion of true tumor regions and reduces missed detections relative to U-Net. This comparison is made against a baseline constrained to an identical training configuration and therefore establishes the effect of the architectural change under that configuration; it does not establish competitiveness with the current state of the art on BraTS 2020, which remains substantially higher (Section 3.2). The published results collected in Table 2 were obtained under different evaluation protocols—different partitions, fully three-dimensional training, ensembling and test-time augmentation—and are not a direct comparison with the values reported here.

Regarding survival prediction, among the four configurations examined, the model combining imaging with age produced the lowest error on this single validation split, with an MAE, RMSE, and median AE of 251.69, 361.78, and 175.69 days, respectively. Compared with the image-only model, the inclusion of age was associated with a reduction in MAE from 279.41 days to 251.69 days. The differences between configurations are small relative to their uncertainty and are not statistically significant after adjustment for multiple testing, so this ordering should not be taken as established; it is specific to this dataset and this validation split and has not been externally validated. An MAE of approximately 252 days corresponds to roughly 57% of the reported median survival of glioblastoma [55] and is not clinically actionable.

However, incorporating resection status—either alone (MAE: 263.36 days) or together with age (MAE: 291.79 days)—was not associated with a reduction in error relative to the model combining imaging with age; the model combining imaging with resection status has an MAE 16.05 days lower than that of the image-only model, but this paired difference is well within sampling uncertainty (95% CI −55.55 to 22.93 days, Holm-adjusted p=1.000). The limited effect of resection status may be attributable to class imbalance, records labeled N/A, and the small validation sample. As set out in Section 4.2, this null result reflects the quality of this particular label in this particular cohort and must not be read as evidence against the prognostic importance of the extent of resection, which is well established in the surgical literature [6,7,8,9]. Accordingly, these results are regarded as a preliminary comparison among feature combinations, and no causal relationship between clinical features and survival is inferred.

Gradient SHAP analysis shows that, when segmentation performance is favorable, attributed regions are largely concentrated on the tumor lesion; in lower-Dice cases, the attribution is more dispersed, suggesting that the model may be affected by indistinct boundaries or surrounding image signals. This indicates that Gradient SHAP serves as an auxiliary tool for examining whether a model’s judgment is grounded in plausible image regions.

An interactive web-based visualization system was developed, integrating patient clinical data, survival predictions, tumor slice range, segmentation metrics, ground-truth and predicted contours, and Gradient SHAP attribution results. Users can switch among patients, survival prediction models, and MRI background modalities, and inspect outputs slice by slice. The system presents integrated results at both patient and slice levels and can serve as a tool for result presentation, comparing cases, and analyzing model errors; however, it is a research prototype: it has not been evaluated with clinical users, has not been validated in a clinical setting, and is not a medical device, and its outputs must not be used as a basis for clinical decisions.

In summary, this study establishes an integrated pipeline spanning tumor segmentation, survival prediction, model explainability, and results visualization. CSFINet improves whole-tumor segmentation relative to an identically trained U-Net baseline, and, among the four configurations compared on the 47 test patients, the model combining imaging with age produced the lowest error, with an MAE of 251.69 days. All results were obtained from a single training run on a single 80:20 patient-level split and have not been externally validated. Note that the survival models were trained on ground-truth masks but evaluated on CSFINet-predicted masks, so their errors include the segmentation error of the first stage; and the survival training runs were not seeded, so those results cannot be reproduced exactly. These results and the visualization system may therefore serve as a preliminary, exploratory reference for brain tumor image analysis and prognostic assessment, but they are not sufficient for clinical application.

### Future Work

This study used age and resection status as candidate clinical features and evaluated their auxiliary value for survival prediction through four ablation experiments. The model combining imaging with age achieves the best overall performance, whereas resection status, whether added alone or together with age, does not improve on the model combining imaging with age. This may be related to class imbalance in resection status, records labeled N/A, and the limited sample size. Future work should collect more complete and evenly distributed resection status data to better establish its actual influence on survival prediction.

Patient survival is jointly influenced by numerous clinical factors, and the tumor mask and age alone are insufficient to fully characterize prognosis. Future work could also incorporate treatment regimen, tumor grade, recurrence, postoperative records, and general health status, evaluating each feature’s influence through more comprehensive ablation experiments.

Glioma prognosis is also closely related to molecular markers; IDH mutation status, MGMT methylation status, and 1p/19q co-deletion may all influence patient survival. Integrating these genetic and molecular markers in future work would help the model learn more comprehensive relationships among imaging features, clinical data, and biomarkers, thereby improving prognostic capability.

Due to hardware constraints, this study adopted a 2D slice-based training strategy for segmentation and extracted three-dimensional spatial features from the binary whole-tumor mask for survival prediction. With greater GPU memory, future work could explore a fully 3D segmentation model or a 3D Transformer architecture to retain more complete inter-slice spatial information and improve boundary and volume estimation.

Finally, model training and validation were conducted primarily on the BraTS 2020 dataset. Future work should perform external validation using BraTS datasets from other years or other institutions, assessing generalizability across different imaging equipment, patient populations, and data sources. Cross-validation or repeated experiments, along with confidence intervals, would establish whether results for the different feature configurations are stable. Validation on larger-scale multicenter data would further improve the reliability and practical value of the model.

Four items follow directly from the limitations set out in Section 4.2. First, repeated *k*-fold cross-validation of both stages, with all tables reported as the mean and standard deviation across folds [49], would establish whether the present ordering of the feature configurations is stable or an artifact of a single split. Second, external validation on the UPenn-GBM [53] and UCSF-PDGM [54] cohorts, and on later BraTS releases [31], would test generalizability across institutions and scanners. Third, the segmentation stage should be brought closer to current practice through fully three-dimensional training, a compound Dice + cross-entropy loss, data augmentation, and a comparison against a properly configured nnU-Net [16,46] and against Transformer-based architectures [25,26]. Fourth, if survival data with event indicators become available, the regression formulation should be replaced by a time-to-event model [59], and the survival model should additionally be trained on CSFINet-predicted masks so that the training and inference conditions match.

## Figures and Tables

**Table 1:** Distribution of resection status, age, and observed survival in the training and test partitions. GTR: gross total resection; STR: subtotal resection; NA: not recorded. IQR: interquartile range.

Resection Status	Training, *n* (%)	Test, *n* (%)	All, *n* (%)	Median Age (IQR)	Median Survival, Days (IQR)
GTR	98 (52.1)	21 (44.7)	119 (50.6)	63.4 (54.2–69.3)	374 (194–558)
STR	7 (3.7)	2 (4.3)	9 (3.8)	61.0 (60.0–64.0)	544 (436–903)
NA (not recorded)	83 (44.1)	24 (51.1)	107 (45.5)	60.9 (53.7–69.0)	346 (172–538)
Total	188 (100)	47 (100)	235 (100)	61.4 (54.2–69.2)	368 (189–578)

**Table 2:** Published BraTS 2020 segmentation results and the present study. Values for the other methods are taken from the cited publications and were obtained under different data partitions, training regimes, and post-processing pipelines; they are provided for context and do not constitute a controlled comparison.

Method	Cohort/Protocol	WT Dice	TC Dice	ET Dice	Notes
3D U-Net (as reported in [25])	BraTS 2020 validation	0.8411	0.7906	0.6876	3D volumetric training
TransBTS [25]	BraTS 2020 validation	0.9009	0.8173	0.7873	CNN + Transformer, with TTA
nnU-Net [46]	BraTS 2020 test	0.8895	0.8506	0.8203	Winning entry, BraTS 2020; ensemble
LATUP-Net [47]	BraTS 2020	0.8841	0.8382	0.7367	Lightweight 3D attention U-Net
CSFINet (this study)	Internal hold-out (n=47)	0.7849	0.7180	0.6814	2D slice-wise; no TTA or ensemble
U-Net baseline (this study)	Internal hold-out (n=47)	0.5084	0.5602	0.5221	Identical training configuration

**Table 3:** Ablation study design for the survival prediction models.

Model	Tumor Mask	Age	Resection Status
Image Only	Yes	–	–
Image + Age	Yes	Yes	–
Image + Resection Status	Yes	–	Yes
Image + Age + Resection Status	Yes	Yes	Yes

**Table 4:** Hyperparameter specification of the segmentation stage (CSFINet) and of the survival prediction stage. PIU: Patch-wise Interaction Unit; FIU: Feature-wise Interaction Unit.

Component	Hyperparameter	Value
CSFINet encoder	Number of stages/channels per stage	5/64, 128, 256, 512, 1024
PIU	Patch size/window size/heads/embedding dimension	4×4, 2×2, 1×1/7/8/256
FIU	Interaction scales/displacement-field settings	5 (15, 30, 60, 120, 240 px)/3×3 conv, 2-channel field, bilinear grid_sample
Decoder	Upsampling scheme/skip fusion	Bilinear + 1×1 conv/concatenation + residual addition
Normalization/activation	Type	BatchNorm2d/ReLU
Segmentation training	Optimizer, learning rate, schedule, weight decay	Adam, $6\times 10^{-4}$, constant, $5\times 10^{-4}$
Segmentation training	Epochs/early-stopping criterion and patience	24/training cross-entropy, 20 (min_delta $10^{-4}$)
Segmentation training	Patient batch/slice batch/gradient accumulation	1/10/yes
Segmentation loss	Multi-class cross-entropy	4 classes
Survival 3D CNN	Blocks/channels/kernel size/pooling	4/1, 16, 32, 64, 128/3/AdaptiveAvgPool3d(1)
Survival clinical MLP	Layer widths/dropout	d→32→16/0.3
Feature fusion	Concatenated feature dimension	144
Survival loss	Huber loss, threshold δ	365 days
Survival training	Optimizer, learning rate, epochs, model-selection rule	Adam, $10^{-4}$, 100, lowest training loss
Both stages	Global random seed	42 (patient-level split)/1337 (segmentation training and evaluation); survival models unseeded

**Table 5:** Hardware and software configuration of the experimental environment.

Item	Specification/Version
Processor (CPU)	Intel Core i7-10700
Memory (RAM)	32GB
Graphics processing unit (GPU)	NVIDIA GeForce RTX 4070 Ti (12GB VRAM)
Operating system (OS)	Windows 10 (64-bit)
Programming language	Python 3.13.1
Deep learning framework	PyTorch 2.7.1
Hardware acceleration libraries	CUDA 11.8/cuDNN 9.1.0

**Table 6:** Segmentation results over the 47 test patients, reported as mean ± standard deviation with 95% BCa bootstrap confidence intervals of the mean. WT: whole tumor; TC: tumor core; ET: enhancing tumor. The paired difference row refers to the whole-tumor Dice.

Model	Region	Mean Dice ± SD (95% CI)	Median Dice (IQR)	Mean Precision ± SD	Mean Recall ± SD
U-Net (baseline, identical training configuration)	WT	0.5084±0.2717 (0.4266–0.5791)	0.6102 (0.3730–0.7238)	0.8676±0.3356	0.3822±0.2273
U-Net (baseline, identical training configuration)	TC	0.5602±0.3139 (0.4643–0.6399)	0.6933 (0.3949–0.7833)	0.8326±0.3257	0.4668±0.2902
U-Net (baseline, identical training configuration)	ET	0.5221±0.2801 (0.4344–0.5931)	0.6337 (0.4040–0.7132)	0.6617±0.2899	0.4827±0.2884
CSFINet	WT	0.7849±0.1937 (0.7125–0.8272)	0.8569 (0.7899–0.8849)	0.8739±0.1978	0.7480±0.1936
CSFINet	TC	0.7180±0.1984 (0.6483–0.7641)	0.7652 (0.6633–0.8577)	0.7110±0.2222	0.7992±0.2103
CSFINet	ET	0.6814±0.1913 (0.6162–0.7272)	0.7329 (0.6106–0.8150)	0.6535±0.1957	0.7609±0.2071
Paired difference (CSFINet − U-Net)	WT	+0.2766 (0.2304–0.3334), $p=3.5\times 10^{-9}$	—	+0.0063 (−0.0601–0.1086)	+0.3658 (0.3257–0.4140)

**Table 7:** Survival prediction results for the different input feature configurations, evaluated on the 47 test patients. Errors are given in days with 95% bootstrap confidence intervals.

Model	MAE ↓	RMSE ↓	Median AE ↓	Pearson *r* ↑	Spearman ρ ↑
Mean predictor (training mean)	313.64 (251.20–401.69)	406.00	262.53	—	—
Age only (linear regression)	297.43 (234.21–380.41)	389.12	238.34	0.283 (−0.004–0.528)	0.242 (−0.049–0.494)
Image Only	279.41 (210.86–376.97)	399.46	180.53	0.305 (0.019–0.544)	0.312 (0.028–0.550)
Image + Age	**251.69** (186.56–336.79)	361.78	175.69	0.471 (0.213–0.668)	0.462 (0.202–0.661)
Image + Resection Status	263.36 (203.60–354.47)	367.90	171.22	0.441 (0.176–0.646)	0.383 (0.108–0.604)
Image + Age + Resection Status	291.79 (234.10–370.82)	374.29	281.37	0.416 (0.146–0.628)	0.278 (−0.010–0.523)

**Table 8:** Case information for BraTS20_199.

Item	Value
True survival	828 days
Predicted survival	354.481 days
Prediction error (predicted − true)	−473.519 days
Absolute error	473.519 days
Age	69.364 years
Resection status	N/A
Dice coefficient	0.909

**Table 9:** Quantitative assessment of Gradient SHAP attribution quality over the 47 test patients. Values are reported as mean ± standard deviation unless otherwise stated.

Measure	Value	Reference/Chance Level
Attribution mass inside the ground-truth mask	0.882±0.100	0.109±0.041 (relative mask area)
Pointing-game accuracy	0.976/0.984 (15 px)	0.109
Deletion AUC (lower is better)	0.161±0.016	0.208±0.025 (random order)
Insertion AUC (higher is better)	0.869±0.086	0.208±0.025 (random order)
Model-parameter randomization test [38]	0.978±0.067→0.611±0.145	Attribution should degrade
Spearman ρ (attribution mass vs. Dice)	ρ=0.405 (p=0.005)	—

## Data Availability

The dataset of the Brain Tumor Segmentation (BraTS2020) is available at https://www.kaggle.com/datasets/awsaf49/brats2020-training-data (accessed on 17 March 2025). The patient identifiers of the training and test partitions used in this study, together with the training configuration files, are provided in the Supplementary Materials.

## Supplementary Materials

The following supporting information can be downloaded at: https://www.mdpi.com/article/10.3390/diagnostics16193210/s1, Section S1: Training and Test Partition; Section S2: Hyperparameter Configuration and Model Definitions; Section S3: Completed TRIPOD/TRIPOD+AI Checklist; Section S4: Completed STROBE Checklist; Section S5: Additional Case Studies and Web-Interface Walkthrough.
