# Supplementary material for: CSFINet-Based Multimodal MRI Image Segmentation and Survival Prediction
Source: Diagnostics (Basel). 2026 Oct 2;16(19):3210. doi: 10.3390/diagnostics16193210 (PMC13664131; doi:10.3390/diagnostics16193210)
Supplement: Supplementary file 1 [file diagnostics-16-03210-s001.zip › diagnostics-4543299-supplementary.pdf]

Supplementary Materials: Supplementary Materials: CSFINet-Based Multimodal MRI Image Segmentation and Survival Prediction

Jia-Lien Hsu * and Pei-Yu Yu

S1. Training and Test Partition

This section documents the exact partition used throughout the study, so that the experiments reported in the main article can be reproduced. The 235 BraTS 2020 subjects with survival labels were divided at the patient level into 188 training and 47 test subjects by a single simple random draw performed once with the fixed global seed 42, as described in Section 2.1.3. The partition was fixed before any model was trained and was used unchanged for both stages and for all four ablation configurations. A machine-readable copy of the two identifier lists is additionally provided as a comma-separated file with the submission.

Table S1. Subject identifiers of the training and test partitions (BraTS 2020 Training release). Identifiers are given in the form BraTS20_Training_NNN

Partition	n	Subject identifiers
Training	188	168, 340, 184, 367, 219, 347, 109, 098, 238, 074, 039, 105, 203, 201, 112, 353, 068, 346, 114, 069, 076, 030, 115, 127, 080, 006, 057, 121, 164, 178, 066, 158, 239, 161, 032, 013, 036, 029, 043, 097, 120, 183, 052, 222, 223, 077, 042, 102, 165, 079, 160, 027, 179, 206, 236, 001, 003, 078, 047, 101, 116, 163, 194, 091, 086, 189, 180, 099, 037, 159, 062, 023, 119, 177, 152, 034, 012, 360, 220, 007, 028, 166, 005, 123, 033, 192, 063, 157, 129, 337, 210, 071, 368, 065, 045, 342, 041, 124, 024, 230, 196, 082, 040, 235, 048, 095, 214, 044, 167, 249, 004, 106, 054, 153, 338, 217, 345, 050, 188, 081, 035, 008, 111, 092, 084, 253, 354, 090, 009, 014, 060, 241, 151, 018, 191, 073, 250, 154, 252, 341, 064, 055, 108, 051, 231, 204, 233, 226, 366, 251, 195, 059, 049, 089, 022, 058, 246, 185, 358, 224, 228, 145, 038, 182, 348, 002, 053, 173, 147, 175, 104, 100, 117, 088, 242, 075, 339, 254, 122, 369, 021, 225, 072, 107, 015, 093, 216, 103
Test (hold-out)	47	070, 227, 218, 010, 128, 110, 359, 094, 174, 016, 356, 056, 229, 031, 351, 170, 020, 113, 357, 025, 011, 096, 181, 247, 026, 087, 221, 085, 019, 169, 067, 046, 162, 126, 199, 190, 017, 255, 215, 187, 186, 125, 061, 240, 363, 083, 118
Total	235	All BraTS 2020 subjects for which a survival label is distributed

S2. Hyperparameter Configuration and Model Definitions

Table 3 in the main text gives the hyperparameters needed to reproduce the two models at the level of detail expected in a methods section. Table S2 below gives the complete configuration actually used, including the values that are ordinarily left implicit, so that the architecture and the training procedure can be reconstructed exactly. The corresponding configuration files and the model definition source are submitted alongside this article. Note the entry for the global random seed: the patient-level split and the two segmentation training runs were seeded (42 and 1337, respectively), but the survival training runs were not seeded.

Table S2. Complete configuration of the segmentation stage (CSFINet and the U-Net baseline) and of the survival prediction stage.

Group	Setting	Value
Input	Modalities / channel order	FLAIR, T1, T1ce, T2 (4 channels)
	Slice size / volume size	240×240 / $240 \times 240 \times 155$
	Intensity normalization	Per-modality, per-patient Z-score, $\epsilon = 1 \times 10^{-8}$
	Data augmentation	None
CSFINet encoder	Number of stages	5
	Channels per stage	64, 128, 256, 512, 1024
	Convolution kernel / stride	3×3 ($\times 2$ per stage) / 1; 2×2 max-pooling
	Normalization / activation	BatchNorm2d / ReLU
PIU	Patch size	4×4, 2×2, 1×1
	Window size	7
	Number of attention heads	8 (PIU); 4, 8, 16 (Swin blocks)
	Embedding dimension / block depth	256 / 1 (PIU); 256, 512, 1024 / 2, 2, 6 (Swin blocks)
FIU	Interaction scales	5 (15, 30, 60, 120, 240 px)
	Displacement-field estimation	3×3 convolution, 2-channel field, bilinear `grid_sample`, zero padding
Decoder	Upsampling operator	Bilinear resize + 1×1 convolution
	Skip-connection fusion	Concatenation (flow estimation); residual addition (warped feature)
	Output layer / number of classes	3×3 convolution ($64 \rightarrow 4$) + BatchNorm2d + ReLU / 4
Segmentation training	Optimizer	Adam
	Initial learning rate / schedule	6×10^{-4} / constant
	Weight decay	5×10^{-4}
	Patient batch size / slice batch size	1 / 10 (gradient accumulation over each patient)
	Number of epochs	24
	Early-stopping criterion / patience	Training cross-entropy / 20 (min_delta 10^{-4})
	Loss function	Multi-class cross-entropy (4 classes)
	Model-selection rule	Lowest training-loss checkpoint
U-Net baseline	Depth / channels per stage	5 / 64, 128, 256, 512, 1024
	Differences from CSFINet	Architecture only; all other settings identical
Survival image branch	Input tensor	$1 \times 128 \times 128 \times 155$ (binary whole-tumor mask)
	3D convolutional blocks / channels	4 / 1, 16, 32, 64, 128
	Kernel size / pooling	3 / AdaptiveAvgPool3d(1)
	Output feature dimension	128
Survival clinical branch	MLP widths	$d \rightarrow 32 \rightarrow 16$ ($d \in \{1, 3, 4\}$)
	Activation / dropout	ReLU / 0.3
Survival fusion and head	Fused feature dimension	144
	Fully connected head	$144 \rightarrow 64 \rightarrow 32 \rightarrow 1$ (image-only: $128 \rightarrow 128 \rightarrow 64 \rightarrow 32 \rightarrow 1$)
Survival training	Optimizer / learning rate	Adam / 10^{-4}
	Number of epochs / model selection	100 / lowest training-loss checkpoint
	Loss function	Huber, $\delta = 365$ days
Reproducibility	Global random seed	42 (patient-level split) / 1337 (segmentation training and evaluation); survival models unseeded
	Deterministic settings	`cudnn.deterministic = True`, `cudnn.benchmark = False`
	Software	Python 3.13.1, PyTorch 2.7.1, CUDA 11.8, cuDNN 9.1.0

S3. Completed TRIPOD/TRIPOD+AI Checklist

The survival prediction component of this study is a multivariable prediction model and is therefore reported in accordance with the TRIPOD statement [1] and its artificial-intelligence extension, TRIPOD+AI [2]. Tables S3 and S4 give the completed checklist against the TRIPOD item numbering, and Table S5 records the topics that TRIPOD+AI adds to TRIPOD.

Table S3. Completed TRIPOD checklist, items 1–10e (development of a prediction model)

Item	Section	Checklist item	Reported in
1	Title	Identify the study as developing a prediction model	Title; Abstract
2	Abstract	Structured summary of objectives, setting, participants, sample size, predictors, outcome, statistical analysis, results and conclusions	Abstract
3a	Introduction	Explain the medical context and rationale for developing the model	Sections 1.1–1.2
3b	Introduction	Specify the objectives, including whether the study describes model development or validation	Sections 1.3, 2.1.3
4a	Methods: source of data	Describe the study design and data source	Sections 2.1, 2.1.1
4b	Methods: source of data	Specify the key study dates	Section 2.1.2; the BraTS 2020 release does not distribute accrual dates
5a	Methods: participants	Specify the key elements of the setting	Sections 2.1.1, 2.1.3
5b	Methods: participants	Describe eligibility criteria	Section 2.1.3: all 235 BraTS 2020 subjects with a survival label
5c	Methods: participants	Give details of any treatments received	Section 2.1.2: only the categorical resection status is distributed
6a	Methods: outcome	Define the outcome and how and when it was measured	Section 2.1.2
6b	Methods: outcome	Report any actions to blind assessment of the outcome	Not applicable: the outcome is a distributed data field
7a	Methods: predictors	Define all predictors and how and when they were measured	Sections 2.4.1, 2.4.2, 2.5
7b	Methods: predictors	Report any actions to blind predictor assessment	Not applicable
8	Methods: sample size	Explain how the study size was arrived at	Section 2.1.3
9	Methods: missing data	Describe how missing data were handled	Section 2.4.2
10a	Methods: analysis	Describe how predictors were handled in the analysis	Sections 2.4.1, 2.4.2
10b	Methods: analysis	Specify the type of model, all model-building procedures and the method for internal validation	Sections 2.5, 2.8.2, 2.8.4
10c	Methods: analysis	For validation, describe how the predictions were calculated	Sections 2.8.2, 2.5
10d	Methods: analysis	Specify all measures used to assess model performance	Sections 3.2, 3.3.1 and 3.3.2
10e	Methods: analysis	Describe any model updating	Not applicable: no model updating was performed

Table S4. Completed TRIPOD checklist, items 11–22

Item	Section	Checklist item	Reported in
11	Methods	Provide details on how risk groups were created, if done	Not applicable: survival is predicted as a continuous value
12	Methods	For validation, identify any differences from the development data	Section 2.5: the train–evaluation mask mismatch
13a	Results: participants	Describe the flow of participants, including the number with and without the outcome	Section 2.1.3; Table 1
13b	Results: participants	Describe the characteristics of the participants, including missing data	Table 1
13c	Results: participants	For validation, show a comparison with the development data	Table 1
14a	Results: development	Specify the number of participants and outcome events in each analysis	188 training / 47 test; Section 2.1.3
14b	Results: development	Report the unadjusted association between each candidate predictor and the outcome	Sections 3.3.3, 3.3.5; Tables 1 and 7 (age-only reference model)
15a	Results: model specification	Present the full prediction model	Section 2.5 of the main article; Section S2 of this file; trained weights provided with the submission
15b	Results: model specification	Explain how to use the prediction model	Section 3.5 of the main article; Section S5 of this file
16	Results: performance	Report performance measures with confidence intervals	Tables 5, 7
17	Results: model updating	Report the results of any model updating	Not applicable
18	Discussion: limitations	Discuss any limitations	Section 4.2
19a	Discussion: interpretation	For validation, discuss the results with reference to performance in the development data	Sections 4.1, 2.5
19b	Discussion: interpretation	Give an overall interpretation, considering objectives, limitations and similar studies	Sections 4.1, 4.2; Section 5
20	Discussion: implications	Discuss the potential clinical use of the model and implications for future research	Sections 4.2, 5.1
21	Other	Provide information about the availability of supplementary resources	Supplementary Materials statement; Sections S1–S5 of this file
22	Other	Give the source of funding and the role of the funders	Funding statement

Table S5. Topics added by TRIPOD+AI to the TRIPOD checklist, and where they are reported

TRIPOD+AI topic	Reported in
Data preparation and pre-processing pipeline	Sections 2.3, 2.4; Section S2
Definition of the analysis unit and of the data partition level	Section 2.1.3 of the main article (patient-level split; no slice-level split)
Handling of class imbalance / outcome distribution	Section 2.8.1: an unweighted multi-class cross-entropy loss is used; no class re-weighting, resampling, or other imbalance handling was applied. The distribution of the survival outcome is given in Section 2.1.2 and Table 1, and the distribution of error across resection-status groups in Section 3.3.3
Model architecture, initialization and computational resources	Section S2; Table 4
Hyperparameter tuning and the data used for it	Section 2.8.4
Random seeds, determinism and run-to-run variability	Section 2.8.3
Fairness and performance in relevant subgroups	Section 3.3.3 (by resection status). No demographic subgroup analysis is possible: BraTS 2020 distributes age but no sex, race or site identifier
Explainability and its evaluation	Sections 2.9, 3.4.2
Open science: protocol and registration	No protocol was registered and no analysis plan was preregistered. The study is a retrospective analysis of a publicly released dataset, as stated in Section 4.2
Open science: data availability	Data Availability statement; Section S1
Open science: code availability	Supplementary Material (configuration files and model definitions)
Patient and public involvement	None; this is a retrospective analysis of a public de-identified dataset
Intended use and risk of the model	Section 4.2; Section 3.5 (research prototype only)

S4. Completed STROBE Checklist

The cohort-description aspects of this study are reported in accordance with the STROBE statement for observational studies [3]. The completed 22-item checklist is given in Tables S6 and S7.

Table S6. Completed STROBE checklist, items 1–12 (cohort studies)

Item	Section	Checklist item	Reported in
1a	Title and abstract	Indicate the study design in the title or the abstract	Abstract
1b	Title and abstract	Provide an informative and balanced summary in the abstract	Abstract
2	Background/rationale	Explain the scientific background and rationale	Sections 1.1–1.2
3	Objectives	State specific objectives, including any prespecified hypotheses	Section 1.3
4	Study design	Present key elements of the study design early in the paper	Sections 2.1, 2.1.3
5	Setting	Describe the setting, locations and relevant dates	Sections 2.1.1, 2.1.2: BraTS 2020 multi-institutional public release
6a	Participants	Give the eligibility criteria and the sources and methods of selection	Section 2.1.3
6b	Participants	For matched studies, give matching criteria	Not applicable
7	Variables	Define all outcomes, exposures, predictors, confounders and effect modifiers	Sections 2.4, 2.1.2
8	Data sources/measurement	Give sources of data and details of methods of assessment	Sections 2.1.1, 2.3, 2.4
9	Bias	Describe any efforts to address potential sources of bias	Sections 2.1.3 (patient-level split), 2.8.4 (test set used once), 2.5
10	Study size	Explain how the study size was arrived at	Section 2.1.3
11	Quantitative variables	Explain how quantitative variables were handled in the analyses	Sections 2.4.1, 2.1.2
12a–12e	Statistical methods	Describe all statistical methods, subgroup analyses, missing data, loss to follow-up and sensitivity analyses	Sections 3.3.1, 2.1.3, 2.4.2

Table S7. Completed STROBE checklist, items 13–22 (cohort studies)

Item	Section	Checklist item	Reported in
13a–13c	Results: participants	Report the numbers of individuals at each stage, reasons for non-participation and, if applicable, a flow diagram	Section 2.1.3; Table 1
14a–14c	Results: descriptive data	Give the characteristics of the study participants, the number with missing data for each variable, and the follow-up time	Table 1; Section 2.1.2
15	Results: outcome data	Report the number of outcome events or summary measures over time	Table 1 (median survival by group); Section 2.1.2
16a–16c	Results: main results	Give unadjusted and adjusted estimates with their precision, report category boundaries, and translate estimates into absolute terms where relevant	Tables 5, 7; Sections 3.3.5, 3.3.6
17	Results: other analyses	Report other analyses done, such as subgroup and sensitivity analyses	Sections 3.3.3 and 3.4.2
18	Discussion: key results	Summarize the key results with reference to the study objectives	Sections 4.1, 5
19	Discussion: limitations	Discuss limitations, taking account of sources of potential bias or imprecision	Section 4.2
20	Discussion: interpretation	Give a cautious overall interpretation considering objectives, limitations, multiplicity of analyses and similar studies	Sections 4.1, 4.2, 3.2.1
21	Discussion: generalisability	Discuss the generalisability of the results	Section 4.2 (single cohort; no external validation)
22	Other information	Give the source of funding and the role of the funders	Funding statement

S5. Additional Case Studies and Web-Interface Walkthrough

Sections S5.1 and S5.2 present the two case studies not shown in Section 3.4—one of lower and one of moderate segmentation quality—using exactly the format of the case retained in the main text. Sections S5.3 and S5.4 give the step-by-step walkthrough of the web-based visualization system, of which Section 3.5 retains only the summary and one representative view.

S5.1. Case 1: BraTS20_070

The patient in this case is 46.551 years of age, with a resection status of gross total resection (GTR). The true survival is 375 days, and the survival predicted by the model combining imaging with age is 402.089 days, an overestimate of 27.089 days relative to the true value. The Dice coefficient of the segmentation result is 0.610, making this a case of lower segmentation performance. The basic information for this case is presented in Table S8.

Table S8. Case information for BraTS20_070

Item	Value
True survival	375 days
Predicted survival	402.089 days
Prediction error (predicted − true)	+27.089 days
Absolute error	27.089 days
Age	46.551 years
Resection status	GTR
Dice coefficient	0.610

Figure S1 shows the slice-level distribution of tumor pixels and the variation in the Dice coefficient for BraTS20_070. The figure indicates that the true tumor region in this case is distributed mainly over two slice ranges, and that the model's predictions broadly correspond to the principal segments in which the tumor appears, showing that the model captures the spatial distribution of the tumor across slices.

In certain slice ranges, however, the number of tumor pixels predicted by the model is lower than that of the ground truth, indicating that the model still underestimates some tumor regions. Overall, the Dice coefficient of 0.610 for this case indicates that the model captures the principal tumor location but that some discrepancy remains in the completeness of the tumor extent.

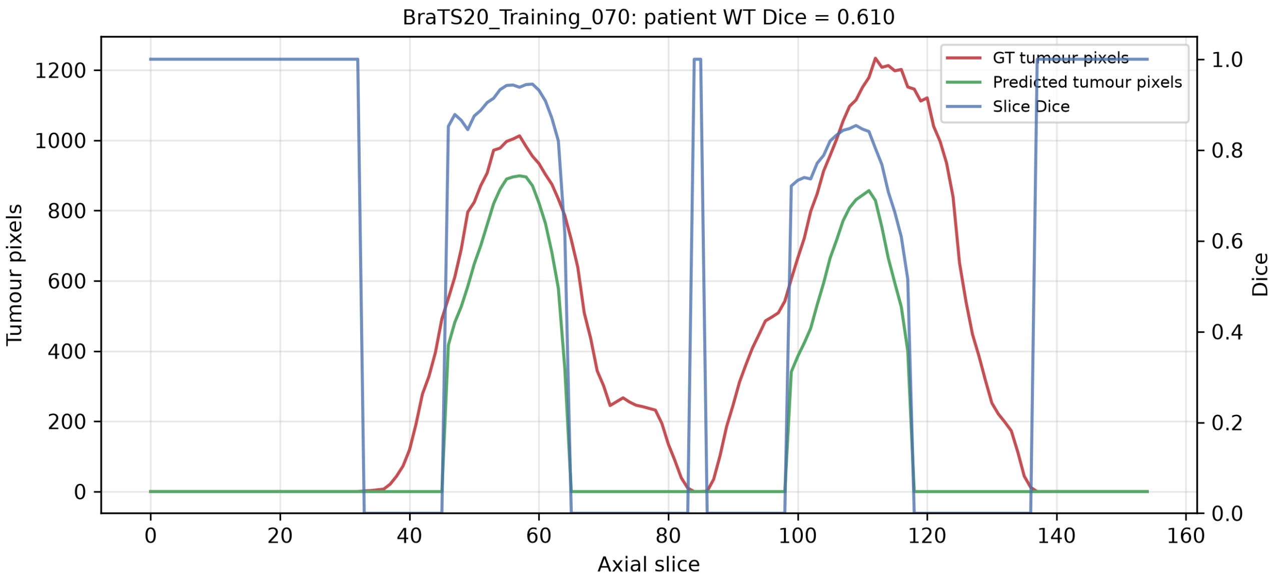

Figure S1. Distribution of tumor pixels and variation in the Dice coefficient for BraTS20_070

Figure S2 shows the segmentation result for slice 54 of BraTS20_070. The left panel shows the ground-truth tumor contour and the right panel the model's predicted contour. The predicted region broadly overlaps the true tumor location and identifies the principal extent of the lesion, indicating good localization capability on this slice.

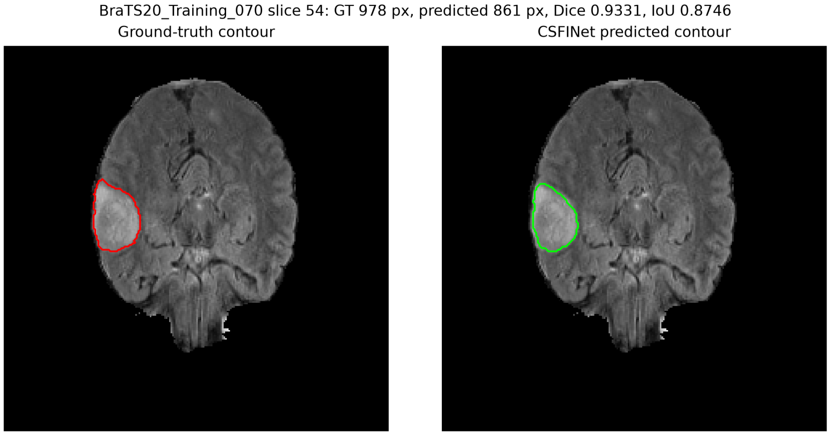

Figure S2. Comparison of segmentation results for slice 54 of BraTS20_070

Figure S3 shows the segmentation result for slice 111 of BraTS20_070. On this slice, the model still predicts the principal tumor location, but the predicted extent is smaller than that of the ground truth, suggesting that the model may be conservative with respect to the tumor boundary or to certain low-intensity regions. This is consistent with the observation in Figure S1 that the predicted pixel count falls below the true pixel count on some slices.

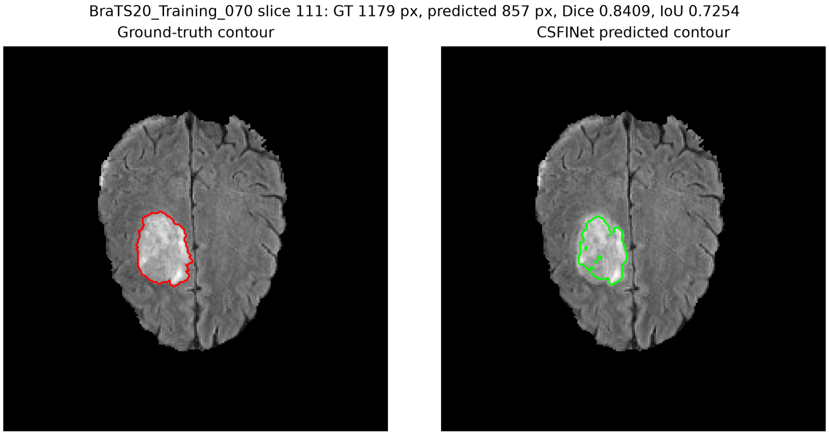

Figure S3. Comparison of segmentation results for slice 111 of BraTS20_070

This case shows that CSFINet localizes the principal tumor region and captures its distribution across slices. Although the predicted extent is smaller than the ground truth on some slices (overall Dice = 0.610), the predicted survival of 402.089 days differs from the true value of 375 days by only 27.089 days. This indicates that, even when the tumor mask does not fully overlap the ground truth, its imaging features combined with age may yield a close survival prediction. However, a single case is insufficient to characterize overall model performance; conclusions must be drawn in conjunction with validation-set metrics.

S5.2. Case 3: BraTS20_094

The patient in this case is 48.773 years of age, with an unknown or unlabeled resection status (N/A). The true survival is 1178 days, and the survival predicted by the model combining imaging with age is 649.400 days, an underestimate of 528.600 days relative to the true value. The Dice coefficient of the segmentation result is approximately 0.735, making this a case of moderate segmentation performance. The basic information for this case is presented in Table S9.

Table S9. Case information for BraTS20_094

Item	Value
True survival	1178 days
Predicted survival	649.400 days
Prediction error (predicted − true)	-528.600 days
Absolute error	528.600 days
Age	48.773 years
Resection status	N/A
Dice coefficient	0.735

Figure S4 shows the slice-level distribution of tumor pixels and the variation in the Dice coefficient for BraTS20_094. The model produces marked mispredictions over certain slice ranges, in particular an elevated predicted pixel count in the vicinity of regions outside the principal ground-truth tumor, indicating that it may misclassify some non-tumor regions as tumor. Moreover, although the predicted curve partially overlaps the ground truth over the principal tumor range, differences remain in the overall extent and trend.

The Dice coefficient of 0.735 for this case indicates moderate overlap between the predicted mask and the ground-truth annotation. Compared with the high-Dice case BraTS20_199 of Section 3.4, BraTS20_094 shows that the model is more prone to false positives or inaccurate extent estimation when the image signal is more complex, the tumor boundary less distinct, or similarly hyperintense regions are present.

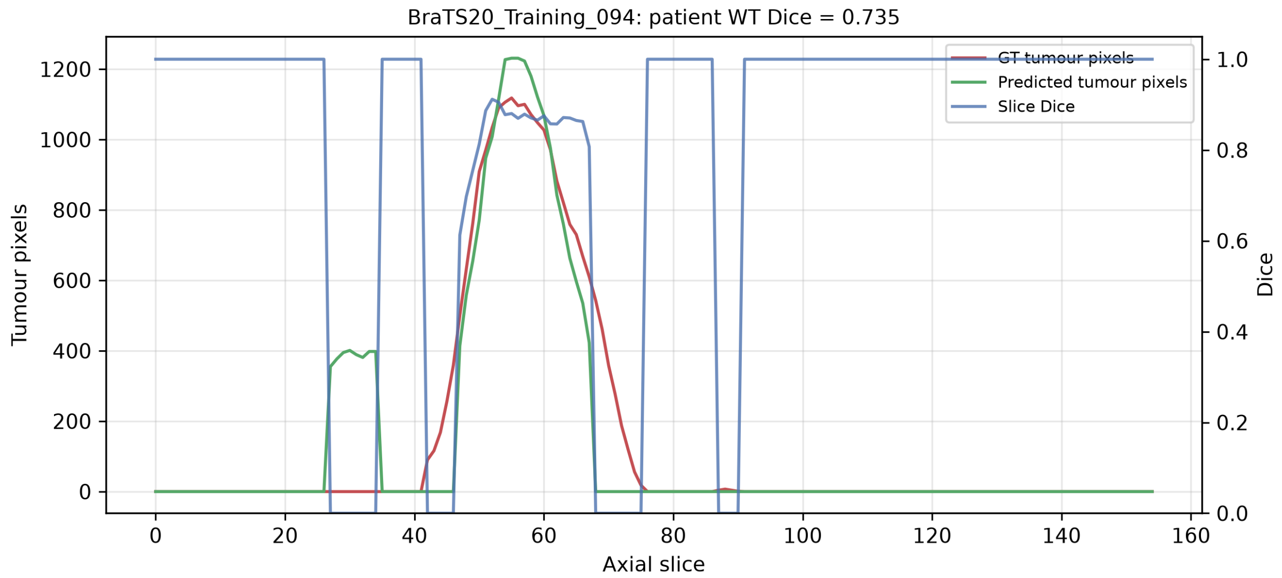

Figure S4. Distribution of tumor pixels and variation in the Dice coefficient for BraTS20_094

Figure S5 shows the segmentation result for slice 57 of BraTS20_094. The left panel shows the ground-truth tumor contour and the right panel the model's predicted contour. The model still detects the principal tumor region, and on this slice the predicted contour follows the ground-truth annotation closely (Dice 0.8790, IoU 0.7842); the predicted region is slightly larger than the annotation (1,223 versus 1,100 pixels) and extends a little beyond it at the margin. The patient-level Dice of 0.735 therefore arises mainly from other slice ranges—in particular the predicted tumor pixels around slices 26–34, where the ground truth contains none—rather than from this slice.

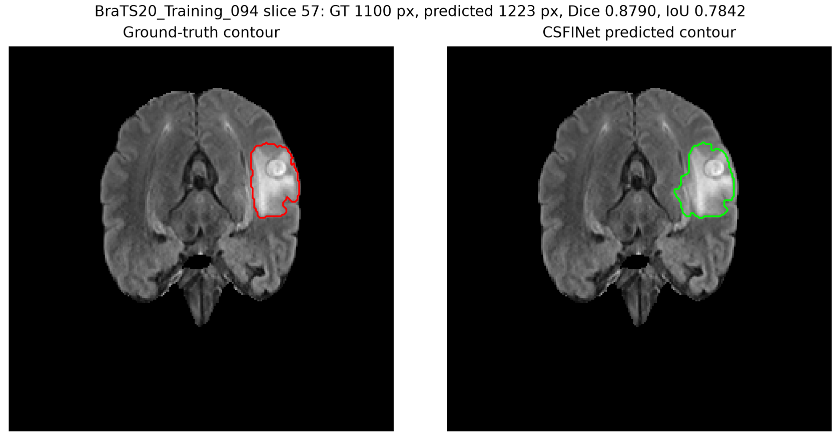

Figure S5. Comparison of segmentation results for slice 57 of BraTS20_094

A Gradient SHAP explainability analysis was additionally performed on slice 57 of BraTS20_094 in order to observe the image regions attended to by the model when segmenting a case of moderate Dice. Figure S6 shows the SHAP attribution results for the GT region and the predicted region, in which red denotes image locations contributing positively to the model's tumor prediction and blue denotes locations with a negative influence on that prediction.

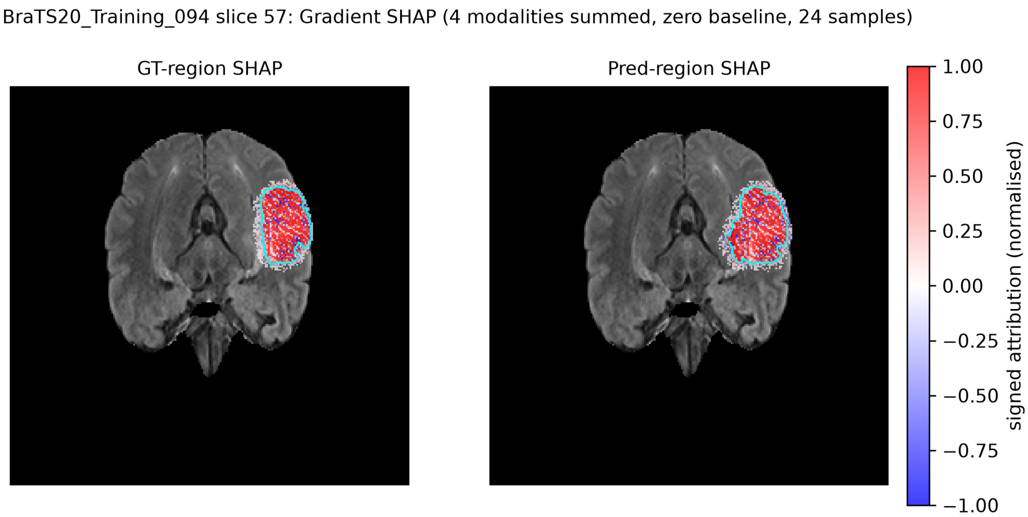

Figure S6. Gradient SHAP explainability analysis for slice 57 of BraTS20_094

The figure shows that the regions of high attribution are concentrated principally at the tumor location, indicating that the model still bases its tumor judgment on the lesion region. The attribution is confined to the lesion much as in the high-Dice case BraTS20_199, so the attribution map of this slice gives no visible indication of the lower patient-level Dice. The relationship between attribution mass and per-patient Dice is quantified over all 47 test patients in Section 3.4.2 and Table 9 of the main article ($\rho = 0.405$, $p = 0.005$).

This case shows that moderate segmentation Dice may mean the predicted tumor mask does not fully reflect the true tumor state. The Gradient SHAP results indicate that the model attends principally to the lesion on the slice examined, so the attribution map does not by itself explain the patient-level segmentation error. The true survival is 1178 days, whereas the predicted value is 649.400 days (an underestimate of 528.600 days), indicating that the tumor mask and age alone remain insufficient to characterize the prognosis of long-surviving patients.

S5.3. Patient Selection, Prediction Data, and Slice Control

As shown in Figure S7, users can select the patient to be analyzed from a drop-down patient menu, whereupon the system loads the corresponding clinical data, survival prediction, tumor slice range, and segmentation results for that patient.

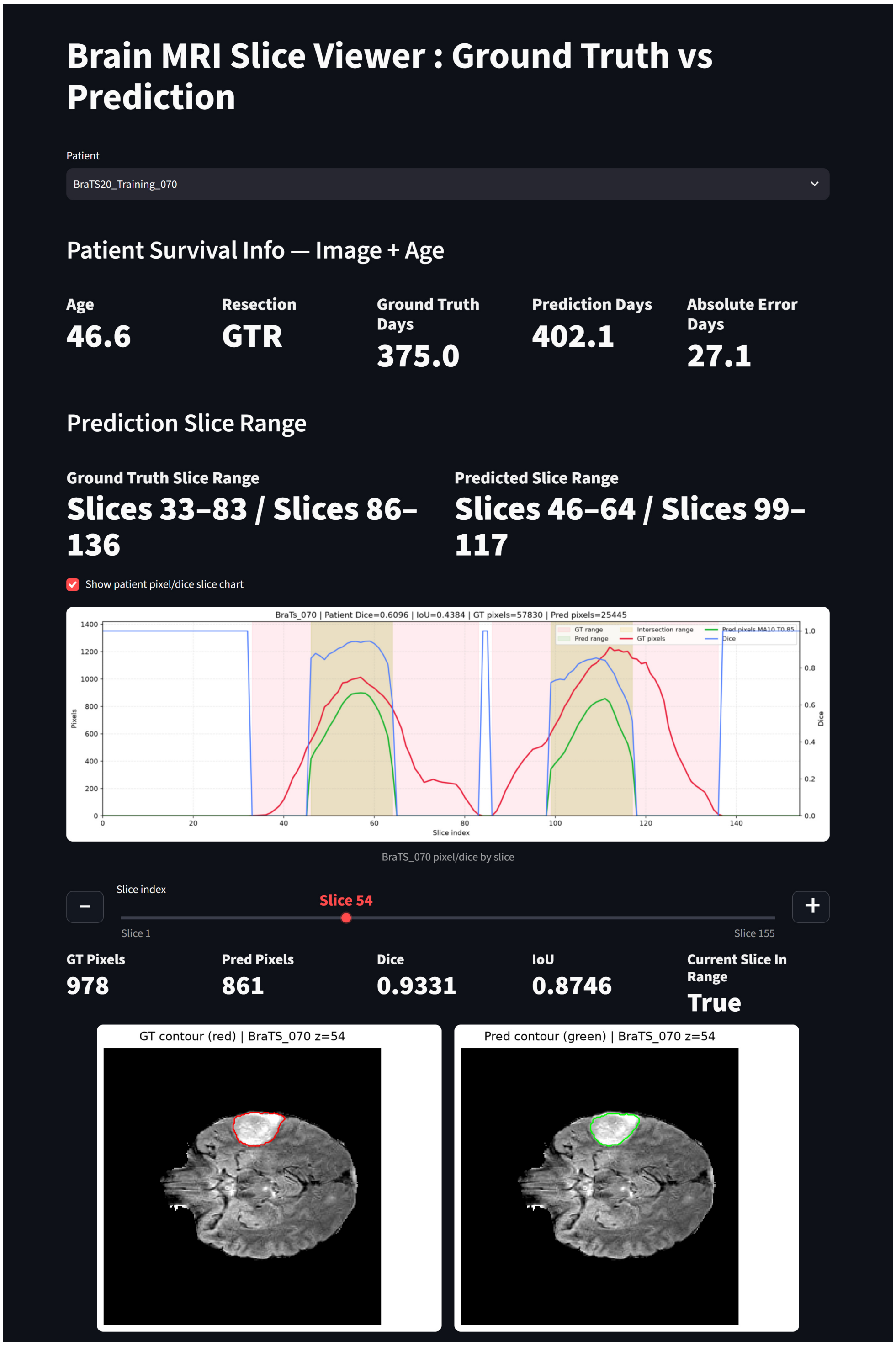

Figure S7. Patient selection, survival prediction information, and slice control interface for BraTS20_Training_070

In reference to Figure S8, the sidebar provides two menus: survival prediction ablation and background image modality. The survival prediction ablation menu allows switching among the four models: image only, image combined with age (Image + Age), image combined with resection status (Image + Resection Status), and image combined with both age and resection status (Image + Age + Resection Status). The system updates the patient's predicted survival and absolute error according to the selected model, facilitating comparison of the predictions across input feature configurations.

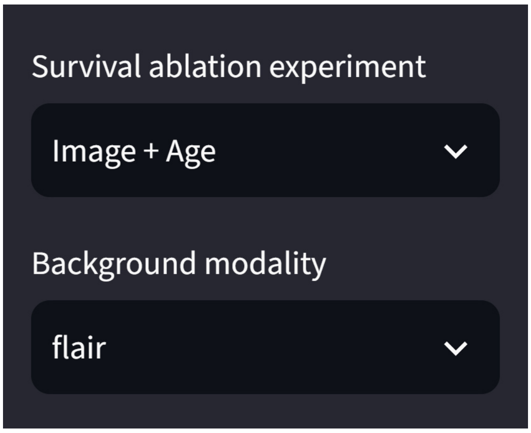

Figure S8. The web-based sidebar: selection menu (ablation study on survival prediction and background imaging modality)

Also in Figure S8, the background image modality menu allows switching among the four MRI modalities FLAIR, T1, T1ce, and T2, using the selected modality as the background image for the tumor contours and Gradient SHAP attribution results. This allows users to observe the tumor imaging characteristics presented by different MRI modalities for the same patient and slice. In this example, the model combining imaging with age (Image + Age) is selected, with FLAIR as the background image modality. The patient is 46.6 years of age, with a resection status of gross total resection (GTR), a true survival of 375.0 days, a model-predicted survival of 402.1 days, and an absolute error of 27.1 days.

The true tumor region of this patient is distributed over slices 33–83 and 86–136, while the tumor region predicted by the model is distributed over slices 46–64 and 99–117. The model identifies the two principal tumor segments, but the predicted extent is narrower than the ground-truth annotation, indicating that some slices at the leading and trailing boundaries of the tumor segments are not identified by the model.

The line chart in the application presents the segmentation results for each slice of the patient. The red curve denotes the number of true tumor pixels in each slice, the green curve the number of predicted tumor pixels in each slice, and the blue curve the Dice coefficient of each slice. When neither the ground-truth annotation nor the model prediction contains any tumor pixel in a given slice, the Dice coefficient of that slice is defined as 1, so that the blue curve takes the value 1 on some tumor-free slices.

Beyond the per-slice results, the chart title also presents the patient-level overall segmentation metrics. Merging the ground-truth and predicted tumor masks over all slices of the patient yields an overall Dice coefficient of 0.6096 and an overall IoU of 0.4384. Across all slices of this patient, the total number of true tumor pixels is 57,830 and the total number of predicted tumor pixels is 25,445.

A slice slider is placed below the line chart, allowing users to move through the axial MRI slices one at a time by dragging the slider or using the buttons on either side. When the slice position changes, the system synchronously updates the tumor pixel count, Dice, IoU, segmentation contours, and Gradient SHAP attribution results for that slice. Slice 54 is selected in this example, for which the number of true tumor pixels is 978, the number of predicted tumor pixels is 861, the Dice coefficient is 0.9331, and the IoU is 0.8746. The Dice and IoU shown here are the evaluation results for slice 54 and are computed over a different scope from the patient-level Dice coefficient and IoU described above.

S5.4. Brain Tumor Segmentation Comparison and Gradient SHAP Visualization

As shown in Figure 15 of the main article (the four-panel slice-54 view), the analysis results for slice 54 are divided into four image panels. The upper-left panel shows the

ground-truth tumor contour (GT contour), marking in red the expert tumor annotation provided in the BraTS 2020 dataset, which serves as the reference against which the model's segmentation is compared.

The upper-right panel shows the model's predicted tumor contour (Pred contour), marking the prediction of CSFINet in green. The true and predicted contours overlap to a high degree over the principal tumor region, although the predicted region is slightly smaller than the ground-truth annotation, consistent with the counts of 978 true tumor pixels and 861 predicted tumor pixels on this slice. The Dice coefficient of this slice is 0.9331 and the IoU is 0.8746, indicating a good degree of segmentation overlap on this slice.

The lower-left panel shows the Gradient SHAP attribution result for the ground-truth tumor region (GT-region SHAP). Taking the ground-truth tumor annotation as the target region, it computes the influence of each input pixel on the mean predicted tumor probability within that region. The cyan contour in the figure denotes the extent of the ground-truth tumor annotation and can be used to observe whether the model's important attribution locations are concentrated within the expert-annotated tumor region and its surroundings.

The lower-right panel shows the Gradient SHAP attribution result for the model's predicted tumor region (Pred-region SHAP). Taking the tumor mask actually predicted by the model as the target region, it computes the influence of each input pixel on the mean predicted tumor probability within that region. The cyan contour in the figure denotes the extent of the tumor predicted by the model and can be used to observe the image locations that give rise to the current segmentation result.

The Gradient SHAP figures in this study use a signed display mode. The program first sums the attribution values of the four MRI input modalities FLAIR, T1, T1ce, and T2, and then normalizes them to the range -1 to 1 according to the distribution of the attribution values of that slice. Red on the color scale denotes positive attribution, indicating that the image features at that location raise the mean predicted tumor probability of the target region relative to the zero-valued baseline image; blue denotes negative attribution, indicating that the image features at that location lower the mean predicted tumor probability of the target region; and white denotes attribution values close to zero, indicating that the net influence of that location on the target region's output is weak. The closer a color is to either end of the scale, the greater the relative strength of the positive or negative attribution.

References

1. Collins, G.S.; Reitsma, J.B.; Altman, D.G.; Moons, K.G.M. Transparent reporting of a multivariable prediction model for individual prognosis or diagnosis (TRIPOD): The TRIPOD statement. *BMJ* **2015**, *350*, g7594. PMID: 25569120, https://doi.org/10.1136/bmj.g7594.
2. Collins, G.S.; Moons, K.G.M.; Dhiman, P.; Riley, R.D.; Beam, A.L.; Van Calster, B.; Ghassemi, M.; Liu, X.; Reitsma, J.B.; van Smeden, M.; et al. TRIPOD+AI statement: Updated guidance for reporting clinical prediction models that use regression or machine learning methods. *BMJ* **2024**, *385*, e078378. PMID: 38626948, https://doi.org/10.1136/bmj-2023-078378.
3. von Elm, E.; Altman, D.G.; Egger, M.; Pocock, S.J.; Gøtzsche, P.C.; Vandenbroucke, J.P. The Strengthening the Reporting of Observational Studies in Epidemiology (STROBE) statement: Guidelines for reporting observational studies. *The Lancet* **2007**, *370*, 1453–1457. PMID: 18064739, https://doi.org/10.1016/S0140-6736(07)61602-X.
